# Hypoxia-induced lncRNA *STEAP3-AS1* activates Wnt/β-catenin signaling to promote colorectal cancer progression by preventing m^6^A-mediated degradation of *STEAP3* mRNA

**DOI:** 10.1186/s12943-022-01638-1

**Published:** 2022-08-19

**Authors:** Li Zhou, Jingwen Jiang, Zhao Huang, Ping Jin, Liyuan Peng, Maochao Luo, Zhe Zhang, Yan Chen, Na Xie, Wei Gao, Edouard C. Nice, Jing-Quan Li, Hai-Ning Chen, Canhua Huang

**Affiliations:** 1 State Key Laboratory of Biotherapy and Cancer Center, West China Hospital, and West China School of Basic Sciences & Forensic Medicine, Sichuan University, and Collaborative Innovation Center for Biotherapy, No. 17, Section 3, South Renmin Rd, Chengdu, 610041 P.R. China; 2 West China School of Basic Medical Sciences & Forensic Medicine, Sichuan University, Chengdu, 610041 P.R. China; 3 Department of Biochemistry and Molecular Biology, Monash University, Clayton, Victoria Australia; 4 Department of Gastrointestinal Oncology Surgery, the First Affiliated Hospital of Hainan Medical University, No. 31, Longhua Road, Haikou, 570102 P.R. China; 5 Colorectal Cancer Center, Department of General Surgery, State Key Laboratory of Biotherapy and Cancer Center, West China Hospital, Sichuan University, No. 17, Section 3, South Renmin Rd, Chengdu, 610041 P.R. China

**Keywords:** Hypoxia, LncRNA *STEAPS-AS1*, STEAP3, m^6^A modification, YTHDF2, Wnt/β-catenin, Colorectal cancer

## Abstract

**Background:**

Hypoxia, a typical hallmark of solid tumors, exhibits an essential role in the progression of colorectal cancer (CRC), in which the dysregulation of long non-coding RNAs (lncRNAs) is frequently observed. However, the underlying mechanisms are not clearly defined.

**Methods:**

The TCGA database was analyzed to identify differential lncRNA expression involved in hypoxia-induced CRC progression. qRT-PCR was conducted to validate the upregulation of lncRNA *STEAP3-AS1* in CRC cell lines and tumor-bearing mouse and zebrafish models under hypoxia. ChIP-qRT-PCR was used to detect the transcriptional activation of *STEAP3-AS1* mediated by HIF-1α. RNA-seq, fluorescent in situ hybridization, RNA pulldown, RNA immunoprecipitation, co-immunoprecipitation, immunofluorescence and immunoblot experiments were used to ascertain the involved mechanisms. Functional assays were performed in both in vitro and in vivo models to investigate the regulatory role of *STEAP3-AS1*/STEAP3/Wnt/β-catenin axis in CRC proliferation and metastasis.

**Results:**

Here, we identified a hypoxia-induced antisense lncRNA *STEAP3-AS1* that was highly expressed in clinical CRC tissues and positively correlated with poor prognosis of CRC patients. Upregulation of lncRNA *STEAP3-AS1*, which was induced by HIF-1α-mediated transcriptional activation, facilitated the proliferation and metastasis of CRC cells both in vitro and in vivo. Mechanistically, *STEAP3-AS1* interacted competitively with the YTH domain-containing family protein 2 (YTHDF2), a N^6^-methyladenosine (m^6^A) reader, leading to the disassociation of YTHDF2 with *STEAP3* mRNA. This effect protected *STEAP3* mRNA from m^6^A-mediated degradation, enabling the high expression of STEAP3 protein and subsequent production of cellular ferrous iron (Fe^2+^). Increased Fe^2+^ levels elevated Ser 9 phosphorylation of glycogen synthase kinase 3 beta (GSK3β) and inhibited its kinase activity, thus releasing β-catenin for nuclear translocation and subsequent activation of Wnt signaling to support CRC progression.

**Conclusions:**

Taken together, our study highlights the mechanisms of lncRNA *STEAP3-AS1* in facilitating CRC progression involving the *STEAP3-AS1*/STEAP3/Wnt/β-catenin axis, which may provide novel diagnostic biomarkers or therapeutic targets to benefit CRC treatment.

**Graphical abstract:**

Hypoxia-induced HIF-1α transcriptionally upregulates the expression of lncRNA *STEAP3-AS1*, which interacts competitively with YTHDF2, thus upregulating mRNA stability of *STEAP3* and consequent STEAP3 protein expression. The enhanced STEAP3 expression results in production of cellular ferrous iron (Fe^2+^), which induces the Ser 9 phosphorylation and inactivation of GSK3β, releasing β-catenin for nuclear translocation and contributing to subsequent activation of Wnt signaling to promote CRC progression.
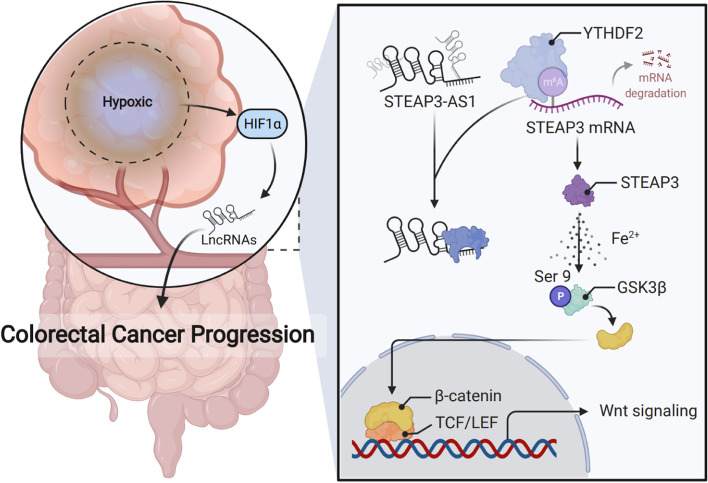

**Supplementary Information:**

The online version contains supplementary material available at 10.1186/s12943-022-01638-1.

## Background

Hypoxia is one of the most common features of solid tumors, which is a driving force for cancer metastasis and is generally caused by an imbalance between rapid proliferation and insufficient angiogenesis [1–3]. In response to hypoxic stress, the transcription factor hypoxia-inducible factor-1α (HIF-1α) is stabilized to transcribe multiple genes involving in cancer cell proliferation, stemness, energy metabolism, metastasis, and drug resistance [4–6]. Mounting studies have revealed the existence of hypoxic fractions in colorectal cancer (CRC) [7–10], the second leading cause of cancer death worldwide [11]. Indeed, hypoxia and the expression of HIFs are closely associated with increased drug resistance and distant metastasis, resulting in poor survival of CRC patients [1]. However, although substantive evidence has supported the regulatory mechanism of HIFs, and many targeted therapeutic strategies have been developed to kill hypoxic tumor cells, lack of well-designed clinical trials has limited their application [12–14]. Therefore, identifying more upstream regulatory factors or downstream effectors of HIFs holds great potential for identifying new diagnostic biomarkers or therapeutic targets which may be of particular scientific significance for treating hypoxic tumors.

Extensive data have demonstrated that hypoxia-induced activation of HIFs also modulates several aspects of epigenetic mechanisms to regulate cancer progression, especially long non-coding RNAs (lncRNAs) [4, 15–17]. LncRNAs are a type of RNA transcripts longer than 200 nucleotides in length, the dysregulation of which has been reported to participate in diverse biological processes in cancer cells, including metabolism, growth and stress response [18–20]. To date, numerous lncRNAs, such as *NEAT1*, *MALAT1*, *MIR31HG*, and *RAB11B-AS1*, have been reported to be activated to promote tumor progression under hypoxic condition [21–23]. For example, lncRNA *RAB11B-AS1* was transcribed by HIF-2 under hypoxia, which enhanced VEGFA and ANGPTL4 expression, promoting angiogenesis and distant metastasis in breast cancer [23]. However, studies focusing on how hypoxia-induced lncRNAs facilitate CRC progression are still limited and their regulatory mechanisms and functions need to be further elucidated.

Antisense lncRNAs are transcribed from the opposite strand of either protein or non-protein coding genes [24]. A growing number of studies are demonstrating that antisense lncRNAs function in several aspects of gene regulation by exerting cis or trans regulation [25, 26]. Indeed, antisense lncRNAs play important roles in many biological processes including cancer initiation and development, mainly through interacting with DNAs, RNAs and proteins [27–29]. For example, a conserved antisense lncRNA, *BDNF-AS*, was reported to negatively regulate its sense transcript both in vitro and in vivo by changing the chromatin structure near the BDNF locus [30]. Another well-studied example of antisense lncRNA in cancer is *HOTAIR* (HOX transcript antisense RNA), which has been demonstrated to promote proliferation, invasion, metastasis, and drug resistance in a number of cancer types, highlighting the great potential of antisense lncRNAs as diagnostic or prognostic indicators for cancer treatment [31–33]. Therefore, it is scientifically important to identify more antisense lncRNA candidates to benefit the development of novel strategies for cancer therapy.

In this context, we searched the TCGA databases to define hypoxia-regulated antisense lncRNAs involved in CRC. Among several candidates, we identified that lncRNA *STEAP3-AS1* was transcriptionally induced by hypoxia, which was aberrantly upregulated in clinical CRC tissues and positively correlated with poor prognosis of CRC patients. Further, we found that lncRNA *STEAP3-AS1* interacted with YTHDF2, thus upregulating mRNA stability of *STEAP3* and consequent STEAP3 protein expression. STEAP3 protein then activated Wnt/β-catenin signaling in an iron-dependent manner, resulting in CRC progression. These findings may unveil a novel pathway for explaining hypoxia-promoted CRC and present potential biomarkers or targets for predicting and treating CRC.

## Material and methods

### Antibodies and reagents

Antibodies used are as follows: anti-STEAP3 (cat# sc-376327, 1:1000 dilution), anti-Axin (cat# sc-293190, 1:500 dilution), anti-p-GSK3β (Ser 9) (cat# sc-373800, 1:1000 dilution) and anti-GSK3β (cat# sc-9166, 1:1000 dilution) were purchased from Santa Cruz Biotechnology; anti-ZO-1 (cat# 8193, 1:1000 dilution), anti-E-cadherin (cat# 3195, 1:1000 dilution), anti-Vimentin (cat# 5741, 1:1000 dilution), anti-Claudin-1 (cat# 13995, 1:1000 dilution), anti-GAPDH (cat# 5174, 1:2000 dilution), anti-β-catenin (cat# 8480, 1:1000 dilution), anti-Histone H3 (cat# 4499, 1:1000 dilution), anti-Snail (cat# 3879, 1:1000 dilution), anti-Slug (cat# 9585, 1:1000 dilution) and anti-HIF-1α (cat# 36169, 1:1000 dilution) were purchased from Cell Signaling Technology; anti-m^6^A (cat# ab208577, 1:1000 dilution), anti-YTHDF1 (cat# ab252346, 1:1000 dilution), anti-YTHDF2 (cat# ab246514, 1:1000 dilution), anti-METTL3 (cat# ab195352, 1:1000 dilution), anti-METTL14 (cat# ab220030, 1:1000 dilution) were purchased from Abcam.

Regents used in this study: Dimethyloxalylglycine (DMOG, cat# S7483) and actinomycin D (Act D, cat# S8964) were obtained from Selleck; DMSO (cat# D2650), Crystal Violet (cat# C0775), MTT (cat# M2128), FeSO_4_ (cat# 215422) and CoCl_2_ (cat# 15862) were obtained from Millipore Sigma. DMOG, actinomycin D and CoCl_2_ were dissolved in DMSO; Crystal Violet, MTT and FeSO_4_ were dissolved in ddH_2_O.

### Cell lines and cell culture

CRC cells (HCT116, RKO, DLD-1, LoVo, SW480, SW620, HT29), the nonmalignant human colon epithelial cell line NCM460 and HEK293T were obtained from ATCC and maintained in Dulbecco’s modified Eagle’s medium (Gibco) supplemented with 10% fetal bovine serum (BI), 100 U/mL penicillin, and 100 μg/mL streptomycin (Invitrogen).

### Patient-derived organoid

The generation of patient-derived organoid was performed as previously described [34]. Briefly, CRC tissue from patients was minced into small fragments (1–3 mm^3^) pieces and digested by collagenase II and TrypLE Express Enzyme at 37 °C. The organoid was then embedded within Matrigel and cultured in 48-well plates supplemented with human complete feeding medium in a humidified incubator with 5% CO_2_ at 37 °C.

### Establishment of stable knockdown and overexpressed CRC cells

Lentiviral vectors or target plasmid were co-transfected into HEK293T cells with packaging vectors. Lentivirus particles were harvested at 24 and 48 h after transfection to infect CRC cells. shRNA plasmids were synthesized, annealed and cloned into pLKO.1 vector. Stable overexpressed gene were generated by using the pCDH expression vectors.

The sequences of shRNA and overexpression primers were as follows: *STEAP3-AS1* shRNA#1, F 5′-CCGGGCACCTTTAAACTGTCCTACACTCGAGTGTAGGACAGTTTAA AGGTGCTTTTTG-3′, R 5′-AATTCAAAAAGCACCTTTAAACTGTCCTACACTCGAG TGTAGGACAGTTTAAAGGTGC-3′; *STEAP3-AS1* shRNA#2, F 5′-CCGGG CTGTTCCGTGGAGCCATTATCTCGAGATAATGGCTCCACGGAACAGCTTTTTG-3′, R 5′-AATTCAAAAAGCTGTTCCGTGGAGCCATTATCTCGAGATAATGGCTCCACGGAA CAGC-3′; *STEAP3-AS1* overexpression primer, F 5′-GAATTCAGACCC AAACCCCAGAGTCAT-3′, R 5′-GGATCCAGAGATGGGACCTCCCTGTGT-3′.

### Western blot

CRC cells were harvest after washing twice with cold PBS. The pellet was resuspended and incubated on ice in lysis buffer for 30 min, and then the lysate was obtained by centrifugation at 12000×g for 10 min. Proteins were separated by SDS-PAGE, transferred onto PVDF membranes, blocked in 5% nonfat milk and then blotted with specific antibodies.

### RT-qPCR

Total RNA was extracted from tissues and cells using Trizol (Invitrogen, USA) and reverse transcribed to cDNA by using a Reverse Transcription Kit (Takara, Dalian, China). The RNA transcripts levels were analyzed using a Bio-rad CFX96 real-time PCR system (Biorad, USA) and normalized to GAPDH. Primers used in RT-qPCR were listed in Supplementary Table S1.

### Cell growth and proliferation assays

Cell viability was detected by adding 5% MTT and incubation at 37 °C for 2 h at 0, 24, 48, 72 and 96 h. The absorbance of each well was measured at 570 nm. All experiments were performed in at least triplicate.

For colony formation assay, 500 cells were planted and maintained in each well of 24 well plates for 2 weeks. The medium was refreshed every 3 days. Colonies were fixed with 4% paraformaldehyde for 1 hour and then stained with 0.1% crystal violet for 30 min and washed with ddH_2_O. The colony numbers of each well were counted.

### Transwell migration and invasion assays

Migration and invasion assays were performed using Transwell chamber system (Corning, USA). For migration assay, 5 × 10^4^ cells were seeded in the upper chamber of an insert with 0.2 ml FBS-free starvation medium, and 0.6 ml culture media with 20% FBS were added outside the chamber in the wells of the plate. For invasion assays, the upper chamber of the insert was pre-coated with Matrigel (Millipore Sigma) before plating cells. After incubation for 48 h, cells were fixed with 4% paraformaldehyde for 1 hour and then stained with 0.1% crystal violet for 30 min. After rinsing with water and airing, migrating or invading cells were imaged and counted using a Leica DM2500 microscope.

### Immunofluorescence

After seeding onto the glass cover slides (WHB scientific, cat# whb-24-cs) and leaving at 37 °C overnight, cells were fixed by 4% formaldehyde, and then permeabilized with 0.3% Triton X-100 and blocked with 5% BSA. After being incubated with indicated primary antibodies (1:100 dilution) at 4 °C overnight, slides were incubated with Alexa Fluor 488/594-conjugated secondary antibodies (1:200 dilution) for 1 hour at room temperature, followed by DAPI staining of the nuclei (Solarbio, cat# C0060, 1:4000 dilution) at room temperature for 10 minutes. Finally, images were captured using confocal laser scanning microscopy (Carl Zeiss Microimaging) in Pub-lab of West China School of Basic Medical Sciences & Forensic Medicine, Sichuan University.

### Immunohistochemistry

Immunohistochemistry was performed following a standard protocol. Briefly, slides were deparaffinized with xylene and ethanol, and the endogenous peroxidase was blocked by 3% H_2_O_2_ for 10 minutes. After being incubated in retrieval buffer and boiled for 3.5 minutes, slides were washed with PBS for 3 times and blocked with 5% normal serum. Then the slides were incubated with primary antibody at 4 °C overnight followed by 60 minutes-treatment of MaxVision HRP solution (MXB Biotechnology, cat# 5020). After being stained with DAB Peroxidase Substrate (MXB Biotechnology, cat# 0031), the antigen levels were detected using EnVision Detection System (Agilent Technologies, K5007).

### Co-immunoprecipitation (co-IP)

After being collected and washed 2 times with pre-cooled PBS, cells were lysed using 1 mL complete protease and phosphatase inhibitor added IP lysis buffer (100 mM NaCl, 20 mM Tris-HCl, pH 7.5, 0.5 mM EDTA, 0.1% NP-40) and incubated on ice for 30 min. After centrifugation at 12000 rpm at 4 °C for 10 min, 80 μL of the supernatant was transferred and mix with 5× lording buffer in a new tube as the control. The remaining supernatant was transferred to a new tube and incubated with 1 μg indicated antibody overnight at 4 °C. After being washed 3 times with IP lysis buffer, 30 μL of protein A/G agarose beads (GE Healthcare, cat# 17-0963-03) were added into the mixture, and rotated for another 2 h at 4 °C. The beads were washed using washing buffer (150 mM NaCl, 0.5 mM EDTA, 20 mM Tris-HCl, pH 7.4, 0.5% NP-40) for 3 times, and proteins were separated by SDS-PAGE loading buffer with 10 min incubation at 100 °C, followed by immunoblotting analysis.

### In vivo orthotopic implantation and spleen injection model

Six to eight weeks old male BALB/c nu/nu mice were used. For orthotopic implantation, 1 × 10^7^ PBS suspended cells was injected subcutaneously. Tumors were collected and sliced into 3 × 3 mm pieces for orthotopic implantation once their diameter reached 1 cm. After being anesthetized and laparotomized, CRC tissues were positioned in the wound and tied down using a suture. The intestines were then placed back followed by closing the peritoneum after sterilization. For the spleen injection model, 5 × 10^5^ cells were injected into the spleen through an incision on the left side of abdomen. Mice were sacrificed at 6–8 weeks after implantation or injection to examine the lung and liver metastases. H&E staining was performed after tissues were fixed in 4% formaldehyde.

### Zebrafish xenograft model

Tg (flk1:eGFP) zebrafishes were used to establish zebrafish xenograft model of human CRC. After being anesthetized with 0.04 mg/mL tricaine (Millipore Sigma), zebrafishes received a microinjection of 200 mCherry stably expressed CRC cells. The tumor cells-bearing zebrafishes were randomly divided into two groups after examination of mCherry fluorescent signal in the next day. Zebrafishes were maintained under normal oxygen or hypoxic conditions for a period of 3 days. Finally, the mCherry fluorescent signal was read to examine the distribution and metastasis of CRC cancer cells using a stereo microscope.

### In situ hybridization (ISH)

In situ hybridization assays were performed to evaluate the *STEAP3-AS1* levels in the CRC xenograft model. Sections were deparaffinized with xylene and ethanol, and the endogenous peroxidase was blocked by 3% H_2_O_2_ for 10 minutes at room temperature. After being incubated with 3% citric acid and freshly diluted pepsin for about 60 s at 37 °C, slides were washed 3 times with PBS and fixed with 1% formaldehyde with addition of 0.1% DEPC for 10 min at room temperature. The sections were then pre-hybridized at 40 °C for 2 hours in a hybrid box with 20 mL 20% glycerinum placed in the bottom. Twenty microlitre hybridization liquid was then added and left at 40 °C overnight. After being washed successively with 2 × SSC, 0.5 × SSC, 0.2 × SSC (15 minutes for each), the sections were blocked with blocking reagent for 30 min at 37 °C. Next, sections were incubated with biotin-digoxigenin for 1 hour at 37 °C followed by Strept Avidin-Biotin Complex (SABC) for 20 min at 37 °C. After being incubated with biotin peroxidase, sections were subjected to DAB. This was followed by hematoxylin redye, dehydration using graded ethanol and vitrification with dimethylbenzene. Sections were analysed using an EnVision Detection System (Agilent Technologies, K5007).

### Fluorescence in situ hybridization (FISH)

After being fixed with 4% formaldehyde, cells were permeabilized with 0.3% Triton X-100 and blocked with 5% BSA. Cells were then pre-hybridized at 37 °C for 30 min followed by incubation with lncRNA FISH Probe Mix for hybridization at 37 °C overnight. After being washed three times, DAPI was added to stain the nucleus. Images were captured at 555 nm using confocal laser scanning microscopy (Carl Zeiss Microimaging).

### RNA immunoprecipitation (RIP)

Cells were harvested for nuclear isolation before incubating with m^6^A antibody for 4 h at 4 °C in 1× immunoprecipitation buffer supplemented with RNase inhibitors. Prewashed protein A/G magnetic beads (30 μL) were added and incubated overnight at 4 °C. After washing 3 times and incubating with proteinase K digestion buffer, RNA was finally extracted using phenol-chloroform and analyzed by qPCR.

### RNA pulldown assay

Briefly, the in vitro biotin-labelled RNAs were transcribed with 10 × Biotin RNA labeling mix (Roche, cat# 1165597910) and T7 enzyme mix (New England Biolabs, cat# M0251S), and heated at 65 °C for 5 min. Samples were cooled to room temperature to form the proper secondary structure in the presence of 10 mM HEPES, 10 mM MgCl_2_ and 0.1 M NaCl. The RNAs were then incubated with Streptavidin Magnetic Beads (Beyotime Biotechnology, cat# P2151) for 15–30 minutes at room temperature with agitation. Protein lysates were then mixed with the RNA-beads complex for 30–60 minutes at 4 °C with agitation or rotation. The pulldown complexes were then washed and boiled at 95–100 °C for 5–10 minutes, followed by immunoblotting.

### Chromatin immunoprecipitation (ChIP) assays

Chromatin immunoprecipitation assay was performed using a ChIP kit (Millipore Corp.) following the manufacturer’s protocol. Firstly, after being cross-linked with 1% formaldehyde, DLD-1 or SW480 cells (1 × 10^7^) were sonicated at 30% maximum power for 8 min (5 s pulse after every 10 s). Supernatants were transferred into a new tube for immunoprecipitation with 1 μg of specific antibodies or IgG antibody after centrifugation at 15000×g for 10 min. The target protein and their binding DNA complex was sedimented using prewashed agarose beads (GE Healthcare, cat# 17–0963-03). After elution and purification, DNA was analyzed by RT-qPCR. Primers used in ChIP-qPCR are listed in Supplementary Table S2.

### TCGA analysis and RNAseq analysis

Gene expression data and the corresponding clinical information were obtained from TCGA repository using the *GDCquery* function of the TCGAbiolinks R package as well as a recent updated clinical data resource [35]. LncRNAs upregulated in CRC were analyzed using DESeq2 R package (log2FoldChange > 0.5 & padj< 0.05). The hypoxia signature score was calculated based on a gene set as previously described [36] using ‘ssgsea’ method of the GSVA R package, and correlations with each lncRNAs were analyzed by Pearson Correlation (coefficient > 0.15 & padj < 0.05). For the survival and kaplan-meier analysis, patients were stratified into two groups (high & low expression) using surv_cutpoint and surv_categorize function of the survminer R package and the progression-free survival was analyzed.

Total RNA was extracted using TRIzol (Invitrogen, Carlsbad, CA, USA). RNA-sequencing analysis was performed at Novogene (Tianjin, China). TCGA data was downloaded and extracted using TCGAbiolinks v.2.22. The rlog transformation and differential expression analysis were performed using DESeq2 v.1.34. The differentially expressed genes (Padj ≤0.05 and log2 (fold change) ≥ 0.5) were subjected to enriched biological pathways analysis using DAVID bioinformatic Resources (2021 Update). Enriched pathways were visualized using clusterProfiler v.4.22 and ComplexHeatmap v.2.10.

### Statistics

All data were from at least three independent experiments and presented as the mean ± SD. The *P* value was calculated using GraphPad (version 9). *P* < 0.05 was considered statistically significant. Comparisons between two groups and repeated measurements over a period of time were performed by two-tailed Student t test, one-way analysis of variance (ANOVA) or two-way ANOVA. Correlation between two independent groups were performed using Pearson’s Chi-square test. Kaplan-Meier method were used to generate survival curves.

## Results

### LncRNA *STEAP3-AS1* is transcriptionally induced by HIF-1α under hypoxia

**Fig. 1:**
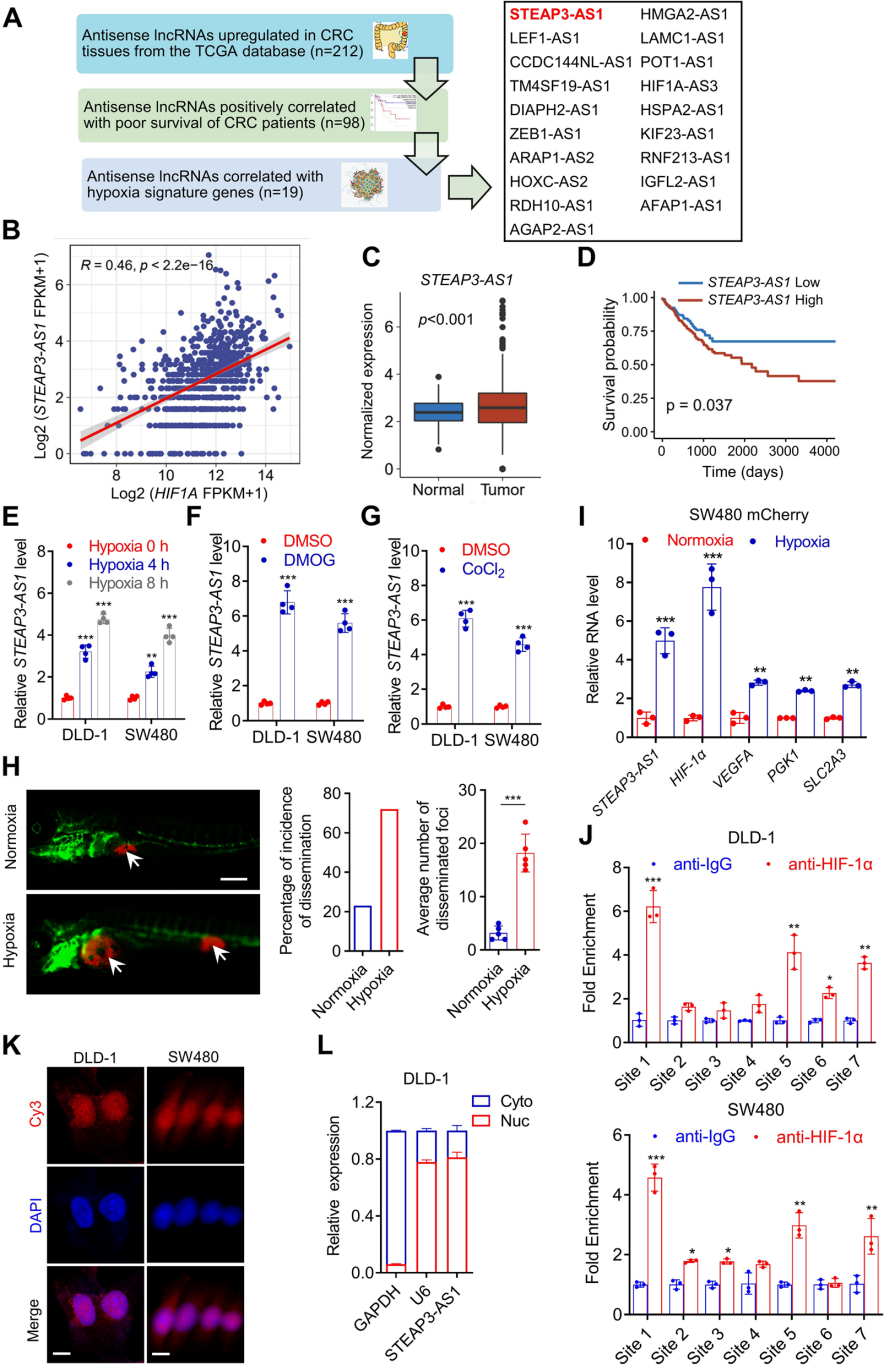
LncRNA *STEAP3-AS1* is transcriptionally induced by HIF-1α under hypoxia. **A** Schematic diagram describing the screening process of candidate antisense lncRNAs using the TCGA dataset. **B** The correlation between *STEAP3-AS1* and *HIF1A* RNA level in TCGA datasets was analyzed by Pearson correlation test. **C** The expression of *STEAP3-AS1* in normal and CRC samples from the TCGA datasets. **D** Kaplan–Meier analysis of progression free survival of CRC patients with low or high *STEAP3-AS1* expression according to the TCGA dataset (*P* = 0.037, log-rank test). **E** qPCR was performed to determine relative *STEAP3-AS1* RNA level in DLD-1 and SW480 cells after treatment with 1% O_2_ for 0 h, 4 h and 8 h. **F**-**G** Relative *STEAP3-AS1* expression in DLD-1 and SW480 cells treated with DMOG (1 mM) or CoCl_2_ (100 μM) for 48 h was determined by qPCR. **H** 200 SW480 cells expressing mCherry were implanted into the perivitelline space of 3dpf flk:eGFP Casper zebrafishes. After being under normoxic or hypoxic (8% O_2_) condition for 3 days, the zebrafishes were then monitored by stereo microscopy. Scale bar: 250 μm. **I** qPCR was performed to determine the relative RNA levels of *STEAP3-AS1, HIF1A, VEGFA, PGK1, SLC2A3* in mCherry SW480-derived zebrafish xenograft models with or without hypoxic treatment. **J** ChIP assay investigating the binding capacity of HIF-1α to each HRE was conducted in DLD-1 and SW480 cells. **K** FISH assay was conducted to determine the subcellular location of lncRNA *STEAP3-AS1* (Cy3) in DLD-1 and SW480 cells. DAPI-stained nuclei are blue. Scale bar: 10 μm. **L** The expression level of lncRNA *STEAP3-AS1* in the subcellular fractions of DLD-1 cells was detected by qPCR. U6 and GAPDH were used as nuclear and cytoplasmic markers, respectively. Data are means ± s.d. and are representative of at least 3 independent experiments. (* *P* < 0.05, ** *P* < 0.01, and *** *P* < 0.001)

To screen for essential lncRNAs that relate to CRC progression under hypoxia, we designed the screening workflow shown in Fig. 1A. Briefly, we analyzed the expression of antisense lncRNAs in the CRC cohort from TCGA database and found 212 candidate lncRNAs that were highly expressed in tumor tissues. The prognostic significance of these lncRNAs were then analyzed, revealing a total of 98 antisense lncRNAs that correlated with poor survival. Finally, we identified 19 hypoxia-related antisense lncRNAs as determined by a correlation analysis with a hypoxic signature as previously described [36] (Fig. 1A). Among these candidates, lncRNA *STEAP3-AS1* was highly correlated with HIF-1α (R = 0.46) and hypoxia signature genes in CRC tissues from TCGA (Fig. 1B and Fig. S1A). Importantly, lncRNA *STEAP3-AS1* was highly expressed in clinical CRC tissues and positively correlated with poor prognosis of CRC patients (Fig. 1C-D), suggesting *STEAP3-AS1* as a potential diagnostic or prognostic biomarker for CRC.

Next, we ascertained the increased expression of *STEAP3-AS1* in response to hypoxic stress. As shown in Fig. 1E-G, *STEAP3-AS1* expression was elevated in hypoxic CRC cells following treatment with 1% oxygen, DMOG, or CoCl_2_ in vitro*.* Consistently, results from qPCR and ISH analysis on DLD-1 xenografts revealed that the levels of *STEAP3-AS1* in the inner regions were significantly higher than those in marginal regions (Fig. S1B-G). To further verify the involvement of *STEAP3-AS1* in hypoxia promoted CRC progression, a zebrafish orthotopic CRC model was built using SW480 mCherry cells. The zebrafishes were maintained in normal or 8% oxygen condition [37]. As shown in Fig. 1H-I, hypoxia promoted the dissemination of CRC cells and upregulated the expression of *STEAP3-AS1*, as well as other downstream hypoxic targets, indicating that *STEAP3-AS1* upregulation may be essential for hypoxia-mediated tumor metastasis. Collectively, these data suggest that *STEAP3-AS1* is upregulated under hypoxic condition and may participate in hypoxia-promoted tumor progression both in in vitro and in vivo CRC models.

To determine whether *STEAP3-AS1* is a direct target of HIF-1α, we explored the characteristics of the genomic locus of *STEAP3-AS1*. Seven putative hypoxia response elements (HREs) were located near *STEAP3-AS1* locus (Fig. S1H). Next, we performed chromatin immunoprecipitation (ChIP) assays to unbiasedly demonstrate the binding of HIF-1α with the predicted HREs in *STEAP3-AS1*. These results suggested that HIF-1α was significantly enriched at HRE 1, 5, and 7 (Fig. 1J). In addition, analysis from coding potential calculator (CPC) and coding potential assessment tool (CPAT) databases both revealed that *STEAP3-AS1* had a low potential for protein encoding (Fig. S1I). Then, the subcellular distribution of *STEAP3-AS1* was determined using fluorescence in situ hybridization (FISH) analysis. The results suggested that lncRNA *STEAP3-AS1* was mainly located in the nucleus (Fig. 1K). The nuclear localization of *STEAP3-AS1* was further validated by the nuclear/cytoplasmic RNA fractionation assay (Fig. 1L and Fig. S1J). Collectively, these findings reveal that lncRNA *STEAP3-AS1* is a direct HIF-1α target gene and mainly located in the nucleus in human CRC.

### LncRNA *STEAP3-AS1* promotes growth of CRC cells both in vitro and in vivo

**Fig. 2:**
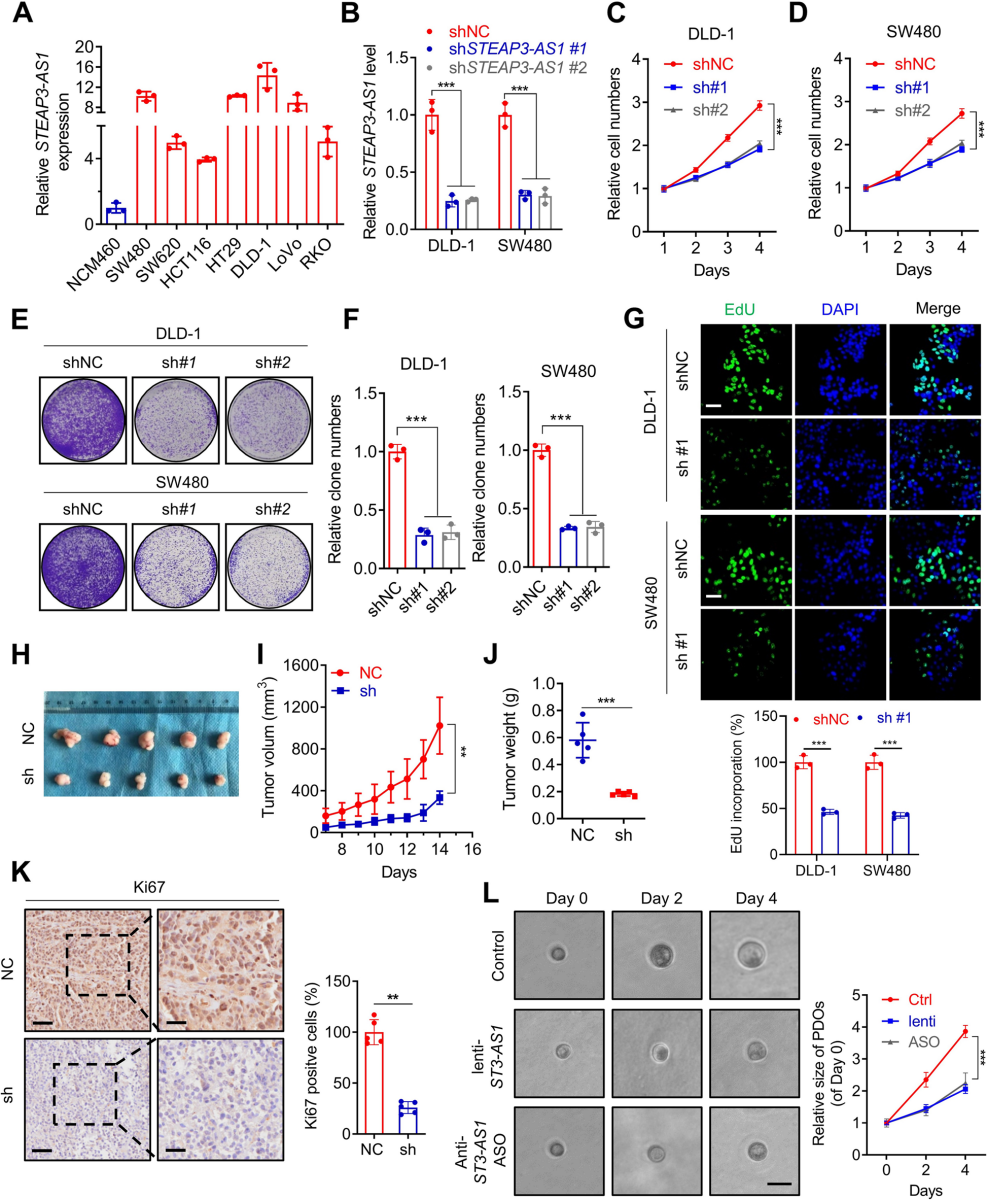
LncRNA *STEAP3-AS1* promotes growth of CRC cells both in vitro and in vivo. **A** Basal level of *STEAP3-AS1* was determined using qPCR assay in the nonmalignant human colon epithelial cell line NCM460 and several CRC cell lines (including SW480, SW620, HCT116, HT29, DLD-1, LoVo, and RKO). **B** qPCR analysis was performed to detect the RNA levels of *STEAP3-AS1* in DLD-1 and SW480 cells with or without *STEAP3-AS1* stable knock-down. **C**-**D** The growth of DLD-1 (**C**) and SW480 (**D**) cells was monitored by MTT assay over a 4-day period with or without *STEAP3-AS1* knockdown. (sh#1, sh*STEAP3-AS1* #1; sh#2, sh*STEAP3-AS1* #2). **E** The effects of lncRNA *STEAP3-AS1* knockdown on the proliferation of SW480 and DLD-1 cells were examined by colony formation assays. Top, DLD-1 cells; bottom, SW480 cells. (sh#1, sh*STEAP3-AS1* #1; sh#2, sh*STEAP3-AS1* #2). **F** Relative clone numbers in (**E**). Left, DLD-1 cells; right, SW480 cells. (sh#1, sh*STEAP3-AS1* #1; sh#2, sh*STEAP3-AS1* #2). **G** EdU assays were used to detect the proliferation rate of SW480 (bottom) and DLD-1 (top) cells with or without *STEAP3-AS1* knockdown. Scale bar: 50 μm. (sh#1, sh*STEAP3-AS1* #1). **H** Images of isolated tumors from the subcutaneous tumor mice model established using DLD-1 cells with or without *STEAP3-AS1* knockdown. **I**-**J** Tumor volume (**I**) and weight (**J**) were significantly decreased in the lncRNA *STEAP3-AS1* knockdown group compared with the control group. **K** Left, representative images of Ki67 staining of the tumor tissue; right, the statistic graph of Ki67 positive cells. Scale bar: left 50 μm, right 20 μm. **L** Left, brightfield images of organoids treated with lenti-sh*STEAP3-AS1* or anti-*STEAP3-AS1* ASO for indicated times; right, the statistic graph of relative size of PDOs. scale bar: 100 μm. Data are means ± s.d. and are representative of at least 3 independent experiments. (** *P* < 0.01 and *** *P* < 0.001)

To study the biological role of *STEAP3-AS1* in CRC, we firstly determined the basal level of *STEAP3-AS1* expression in several CRC cell lines (Fig. 2A). Subsequent viability and colony formation assay suggested that stably knocked down *STEAP3-AS1* expression could clearly attenuate proliferative capability in CRC cells (Fig. 2B-F and Fig. S2A-B). In contrast, overexpression of *STEAP3-AS1* significantly accelerated cell proliferation rate (Fig. S2C-E). This effect of *STEAP3-AS1* on promoting proliferation was further evidenced by EdU staining assay (Fig. 2G and Fig. S2F). To further ascertain the function of *STEAP3-AS1* in CRC proliferation, a subcutaneous xenograft model was established. Consistent with the in vitro results, *STEAP3-AS1* knock down significantly decreased tumor weight and tumor volume compared with those in the control group (Fig. 2H-J). Moreover, the protein level of Ki67, a proliferative maker was detected using immunohistochemistry (IHC) assays. The results confirmed that *STEAP3-AS1* knocked down caused reduction in Ki67 expression (Fig. 2K). In addition, we also used a patient-derived organoid (PDO) model to further verify the function of *STEAP3-AS1*. As shown in Fig. 2L, infection with lenti-sh*STEAP3-AS1* or treatment of antisense oligo targeting *STEAP3-AS1* significantly suppressed the growth of PDOs, indicating the important role of *STEAP3-AS1* in promoting CRC progression. Taken together, these results indicate that lncRNA *STEAP3-AS1* promotes CRC cell proliferation both in vitro and in vivo.

### LncRNA *STEAP3-AS1* facilitates migration and invasion of CRC cells both in vitro and in vivo

**Fig. 3:**
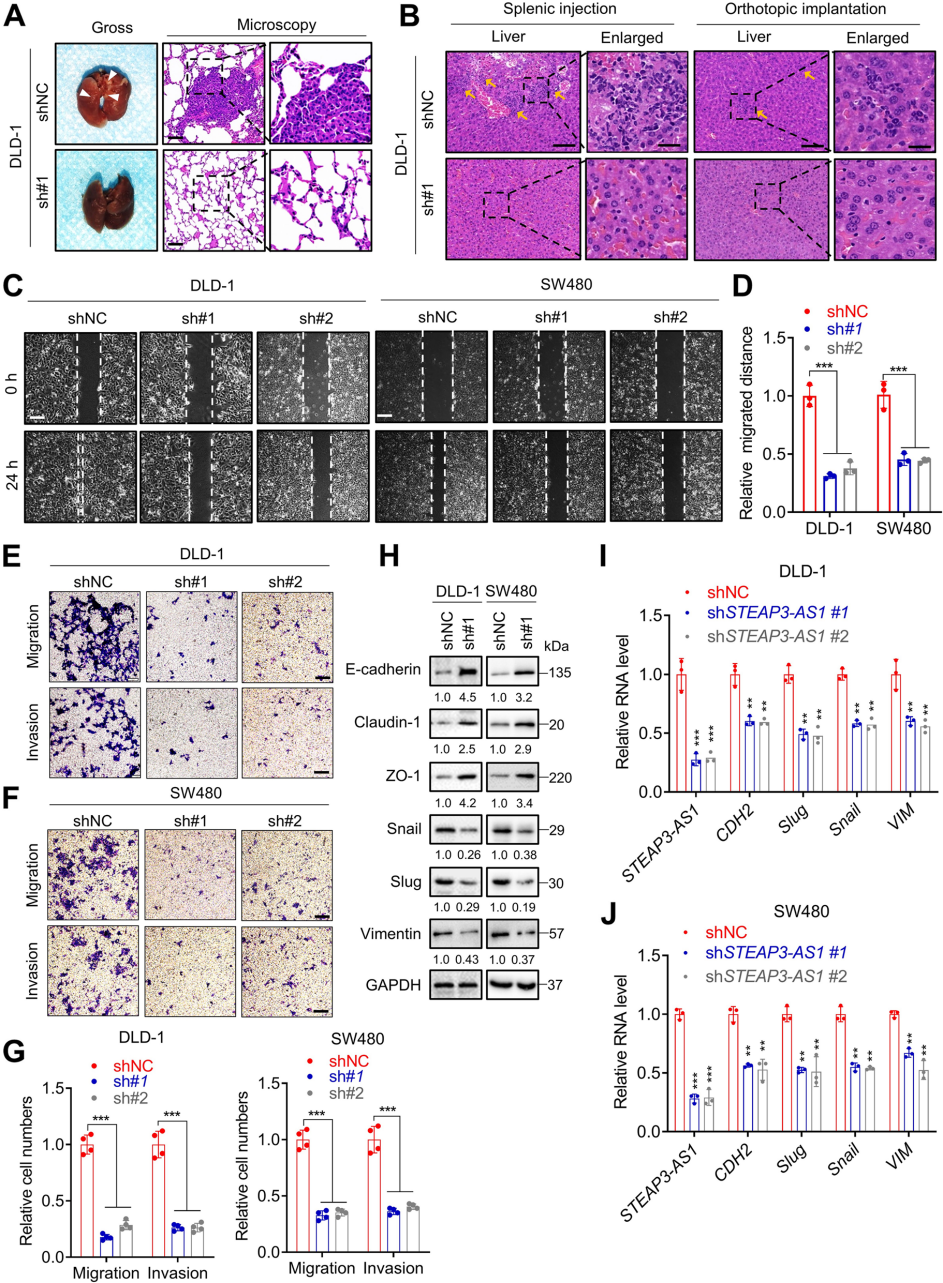
LncRNA *STEAP3-AS1* facilitates migration and invasion of CRC cells. **A** Representative images of lung (left) and their H&E staining (right) from the tail vein injection model using DLD-1 cells. Scale bar: 100 μm. (sh#1, sh*STEAP3-AS1* #1). **B** The metastatic liver colonization model was generated by inoculating DLD-1 cells into the splenic of BALB/c mice (left), or orthotopically implanting CRC xenograft into the cecum of BALB/c mice (right). Representative H&E images of livers staining are shown. Scale bar: left 100 μm, right 25 μm. (sh#1, sh*STEAP3-AS1* #1). **C** Wound healing assay showing cell migration of DLD-1 (left) and SW480 (right) cells with or without *STEAP3-AS1* knockdown. Scale bar: 100 μm. (sh#1, sh*STEAP3-AS1* #1; sh#2, sh*STEAP3-AS1* #2). **D** The statistic graph of relative migration distance in (**C**). (sh#1, sh*STEAP3-AS1* #1; sh#2, sh*STEAP3-AS1* #2). **E**-**F** Migration and invasion of DLD-1 (**E**) and SW480 cells (**F**) with or without *STEAP3-AS1* knockdown were evaluated using transwell assays. Scale bar: 100 μm. (sh#1, sh*STEAP3-AS1* #1; sh#2, sh*STEAP3-AS1* #2). **G** The statistic graph of relative migration cell numbers in (**E-F**). Left, DLD-1 cells; Right, SW480 cells. (sh#1, sh*STEAP3-AS1* #1; sh#2, sh*STEAP3-AS1* #2). **H** EMT markers were detected by western blot in DLD-1 and SW480 cells with or without *STEAP3-AS1* knockdown. (sh#1, sh*STEAP3-AS1* #1). **I**-**J** qPCR analysis of the expression of EMT markers in *STEAP3-AS1* knockdown or control DLD-1 (**I**) and SW480 (**J**) cells. Data are means ± s.d. and are representative of at least 3 independent experiments. (** *P* < 0.01 and *** *P* < 0.001)

We then investigated the role of *STEAP3-AS1* in the mobility of CRC cells. To this end, we firstly constructed tail vein injection models using DLD-1 cells. Following sacrificed, their lungs were isolated for further investigation of the metastatic nudes. Hematoxylin and eosin (H&E) staining results suggested that lncRNA *STEAP3-AS1* deficiency remarkably attenuated the number of lung metastatic nodes (Fig. 3A and Fig. S3A). Furthermore, two metastatic liver colonization models were established by inoculating DLD-1 cells into the spleens of BALB/c nude mice or orthotopically implanting DLD-1-derived CRC xenograft into the cecum of BALB/c nude mice (Fig. S3B). H&E staining of liver isolated from these two models also revealed that knockdown of lncRNA *STEAP3-AS1* decreased the number of liver metastatic lesions compared with those in the control group (Fig. 3B). In vitro experiments including wound healing and transwell assays were performed to demonstrate the role of lncRNA *STEAP3-AS1* on cell mobility of CRC cells. Consistently, knockdown of lncRNA *STEAP3-AS1* significantly suppressed CRC cell migration and invasion (Fig. 3C-G), while overexpression of it increased the migratory and invasive abilities of CRC cells (Fig. S3C-D). Next, the expression of several epithelial-to-mesenchymal transition (EMT) markers was tested, as it is well documented that EMT is one of the hallmarks of elevated cell mobility. Results from western blotting and qPCR analysis revealed that *STEAP3-AS1* knockdown up-regulated the epithelial markers E-cadherin, claudin-1 and Zona occludin-1 but suppressed the mesenchymal markers Snail, Slug and vimentin in DLD-1 and SW480 cells (Fig. 3H-J). However, overexpression of *STEAP3-AS1* could up-regulate the mesenchymal markers but suppress the epithelial markers (Fig. S3E). This was further validated by immunofluorescence (IF) assay, in which the fluorescence intensity of ECAD was distinctly enhanced with *STEAP3-AS1* knockdown (Fig. S3F). Taken together, these data show that lncRNA *STEAP3-AS1* facilitates tumor metastasis in CRC cells both in vitro and in vivo*.*

### LncRNA *STEAP3-AS1* positively regulates STEAP3 to promote CRC progression

**Fig. 4:**
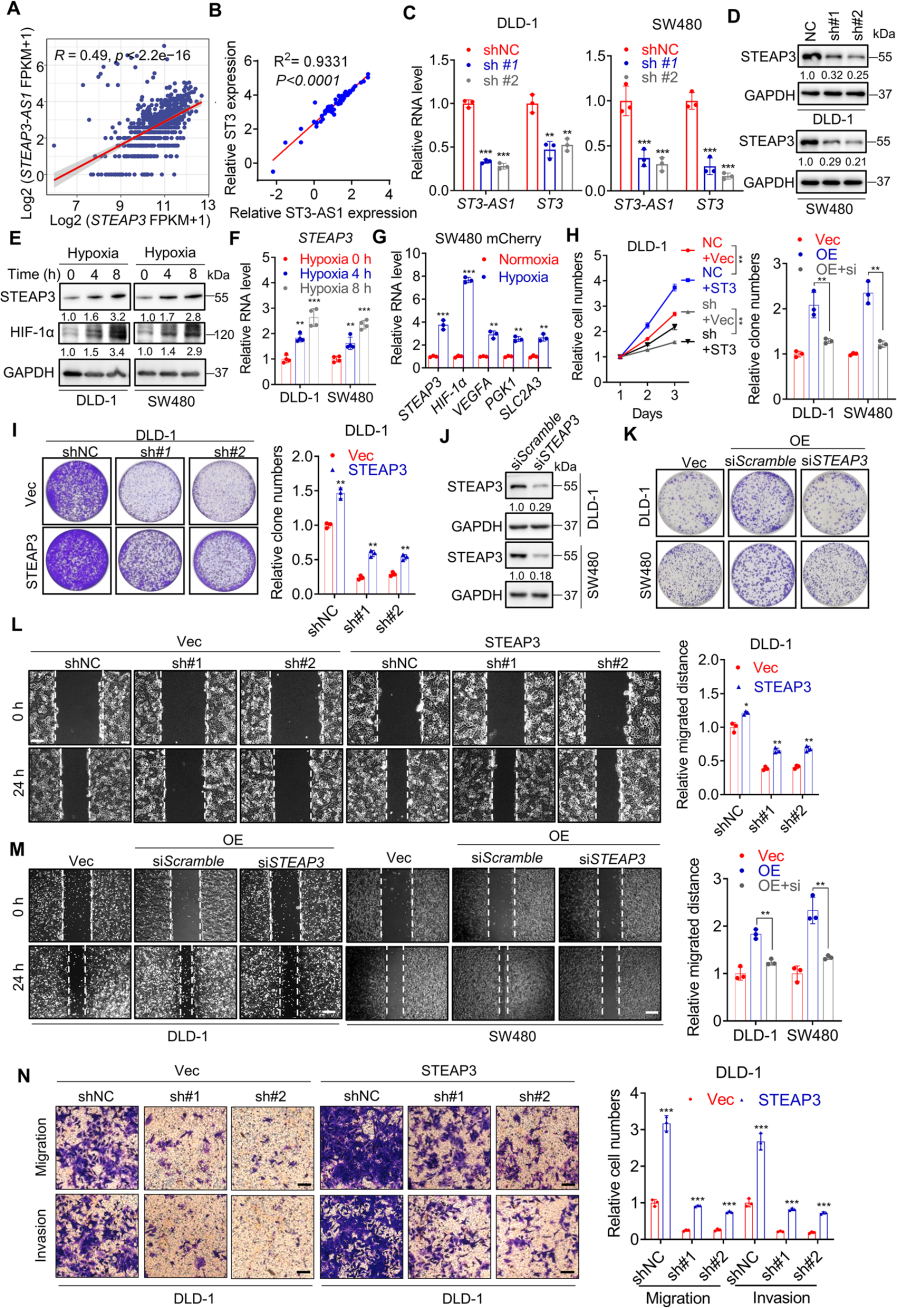
LncRNA *STEAP3-AS1* positively regulates STEAP3 to promote CRC progression. **A**-**B** Correlation analysis of relative RNA level of *STEAP3* and *STEAP3-AS1* in TCGA datasets (**A**) and CRC cell lines from CCLE (**B**), r = Pearson’s correlation coefficient. **C** Relative RNA level of *STEAP3-AS1* and *STEAP3* in control or *STEAP3-AS1*-knockdown DLD-1 (left) and SW480 (right) cells determined by qPCR. (sh#1, sh*STEAP3-AS1* #1; sh#2, sh*STEAP3-AS1* #2). **D** The expression level of STEAP3 protein in control or *STEAP3-AS1*-knockdown DLD-1 and SW480 cells was measured by western blotting. (sh#1, sh*STEAP3-AS1* #1; sh#2, sh*STEAP3-AS1* #2). **E**-**F** The protein levels of STEAP3 and HIF-1α (**E**), and relative RNA level of *STEAP3* (**F**) in DLD-1 and SW480 cells under hypoxia at indicated time points. **G** qPCR analysis of relative RNA levels of *STEAP3*, *HIF-1α* and HIF-1α target genes in mCherry SW480-derived zebrafish xenograft models under normoxia or hypoxia. **H-I** Relative cell numbers at serial time points (**H**) and colony formation (**I**) of control and *STEAP3-AS1*-knockdown DLD-1 cells with or without replenishment of STEAP3. (sh#1, sh*STEAP3-AS1* #1; sh#2, sh*STEAP3-AS1* #2; OE, *STEAP3-AS1* overexpression). **J** The efficiency of siRNA-mediated STEAP3 knockdown was measured by western blotting. **K** The colony formation of control and *STEAP3-AS1*-overexpressing DLD-1 and SW480 cells treated with or without STEAP3 siRNA. (OE, *STEAP3-AS1* overexpression). **L** Wound healing assay (left) and relative migration distance (right) of control and *STEAP3-AS1*-knockdown DLD-1 cells with or without reintroduction of STEAP3. Scale bar: 100 μm. (sh#1, sh*STEAP3-AS1* #1; sh#2, sh*STEAP3-AS1* #2). **M** Wound healing assay (left) and relative migration distance (right) of control and *STEAP3-AS1*-overexpressing DLD-1 cells treated with or without STEAP3 siRNA. Scale bar: 100 μm. (OE, *STEAP3-AS1* overexpression). **N** Migration and invasion of control and *STEAP3-AS1*-knockdown DLD-1 cells with or without reintroduction of STEAP3. Scale bar: 100 μm. (sh#1, sh*STEAP3-AS1* #1; sh#2, sh*STEAP3-AS1* #2). Data are means ± s.d. and are representative of at least 3 independent experiments. (* *P* < 0.05, ** *P* < 0.01, and *** *P* < 0.001)

Since most antisense lncRNAs exert their biological function by regulating their neighboring genes, we examined whether lncRNA *STEAP3-AS1* has an effect on STEAP3 expression. Bioinformatic analysis revealed that the mRNA level of STEAP3 was positively correlated with *STEAP3-AS1* expression both in CRC tissues from the TCGA datasets and CRC cell lines from the cancer cell line encyclopedia (CCLE) (Fig. 4A-B). Consistently, the mRNA (Fig. 4C) and protein (Fig. 4D) levels of STEAP3 were both decreased in *STEAP3-AS1*-knockdown DLD-1 and SW480 cells. Given that *STEAP3-AS1* is transcriptionally induced by HIF-1α under hypoxia, we wondered whether the expression of STEAP3 is responsive to hypoxic stimuli. The data showed that the protein (Fig. 4E, S4A-B) and mRNA (Fig. 4F, S4A-B) levels of STEAP3 were significantly upregulated in CRC cells under hypoxia induced by 1% oxygen, DMOG, or CoCl_2_. Furthermore, the expression of *STEAP3* was obviously increased in SW480 mCherry cells under hypoxia (as indicated by the upregulation of *HIF-1α* and HIF-1α target genes) (Fig. 4G), as well as in the inner region of tumors from DLD-1 xenografts (Fig. S4C-D). In conclusion, these results suggest that *STEAP3* is a hypoxia-induced gene positively regulated by *STEAP3-AS1*.

To further verify the regulatory role of STEAP3 in *STEAP3-AS1*-induced CRC proliferation, cell growth rate (Fig. 4H and Fig. S4E) and colony formation ability (Fig. 4I-K and Fig. S4F) were analyzed in *STEAP3-AS1*- and STEAP3-manipulated CRC cells. As shown in Fig. 4H-K and Fig. S4E-F, overexpression of STEAP3 could partly reverse the decreased cell proliferation induced by *STEAP3-AS1* knockdown in CRC cells and STEAP3 knockdown could suppress cell proliferation promoted by *STEAP3-AS1* overexpression. Consistently, replenishment of STEAP3 could also rescue the attenuated migration and invasion in *STEAP3-AS1*-knockdown CRC cells, as determined by wound healing (Fig. 4L and Fig. S4G-H) and cell migration/invasion assays (Fig. 4N and Fig. S4I). In contrast, knockdown of STEAP3 in *STEAP3-AS1*-overexpressing CRC cells displayed the opposite phenotype (Fig. 4M and Fig. S4J-K). Taken together, these results demonstrate that STEAP3 is responsible for *STEAP3-AS1*-mediated CRC progression.

### *STEAP3-AS1* binds to YTHDF2 to prevent m^6^A-mediated degradation of *STEAP3* mRNA

**Fig. 5:**
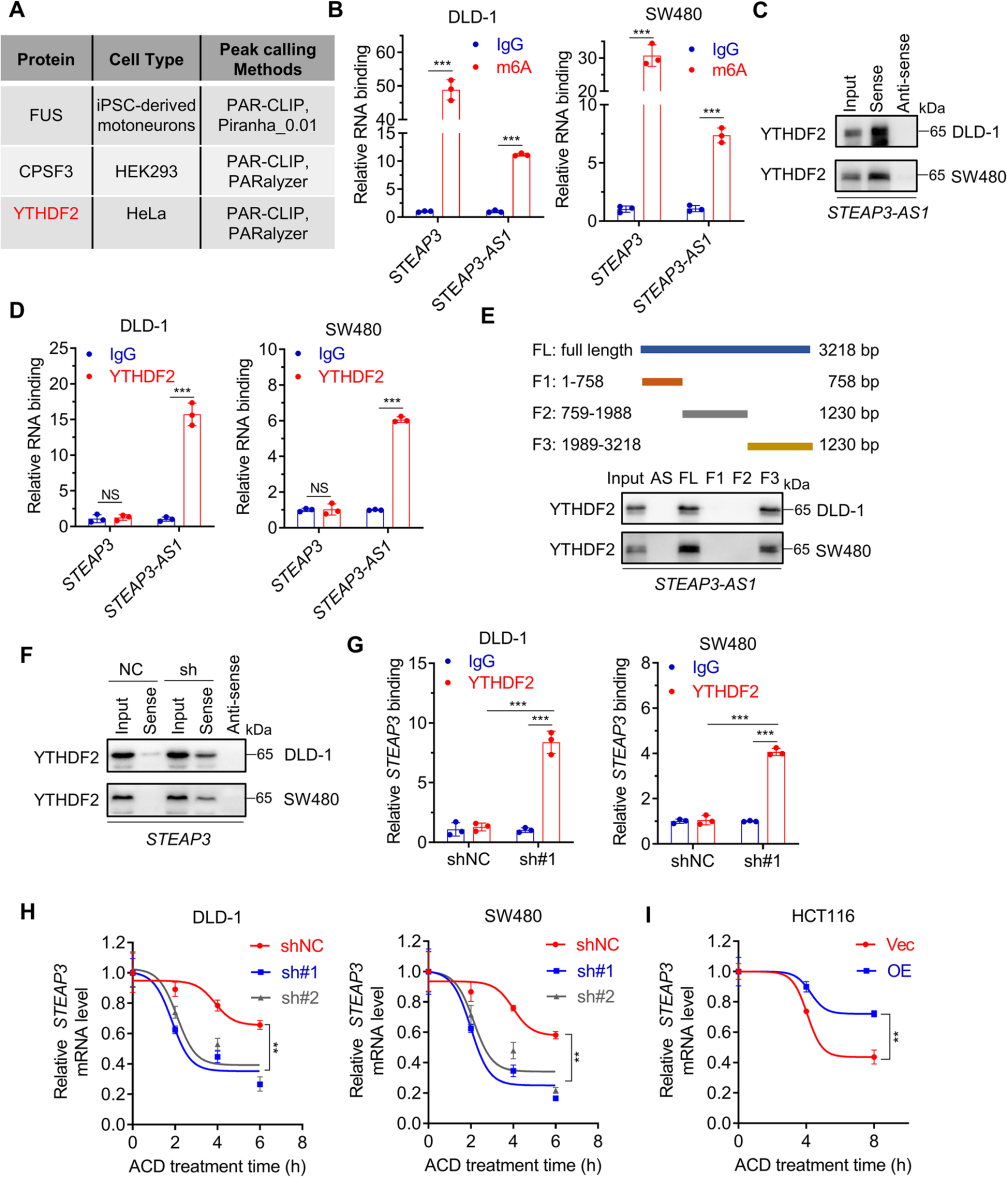
*STEAP3-AS1* binds to YTHDF2 to prevent m^6^A-mediated degradation of *STEAP3* mRNA. **A** The potential binding proteins of *STEAP3-AS1* predicted by the AnnoLnc2 database. **B** m^6^A modification of *STEAP3* mRNA and *STEAP3-AS1* in SW480 and DLD-1 cells analyzed by MeRIP. **C** The interaction between YTHDF2 and *STEAP3-AS1* in DLD-1 and SW480 cells as measured by RNA pulldown. Anti-sense was used as a negative control. **D** RIP analysis of the binding of YTHDF2 to *STEAP3* mRNA and *STEAP3-AS1* in DLD-1 and SW480 cells. **E** The binding of YTHDF2 to indicated truncated *STEAP3-AS1* in SW480 and DLD-1 cells measured by RNA pulldown. **F** RNA pulldown analysis of the interaction between YTHDF2 and *STEAP3* mRNA in control or *STEAP3-AS1*-knockdown DLD-1 and SW480 cells. **G** RIP analysis of the interaction between YTHDF2 and *STEAP3* mRNA in DLD-1 and SW480 cells with or without *STEAP3-AS1* knockdown. (sh#1, sh*STEAP3-AS1* #1). **H** Relative mRNA level of *STEAP3* in control or *STEAP3-AS1*-knockdown DLD-1 and SW480 cells treated with actinomycin D (2.5 μM) at indicated time points. (sh#1, sh*STEAP3-AS1* #1; sh#2, sh*STEAP3-AS1* #2). **I** Relative *STEAP3* mRNA level in control or *STEAP3-AS1*-overexpressing HCT116 cells treated with actinomycin D (2.5 μM) at indicated time points. (OE, *STEAP3-AS1* overexpression). Data are means ± s.d. and are representative of at least 3 independent experiments. (** *P* < 0.01 and *** *P* < 0.001, NS, not significant)

To further dissect the mechanism underlying the regulatory effect of *STEAP3-AS1* on *STEAP3* mRNA, we predicted the potential binding proteins of *STEAP3-AS1* using the AnnoLnc2 database (Fig. 5A). Among the putative binding targets, a reader for RNA N^6^-methyladenosine (m^6^A) modification, YTHDF2, was of particular interest for further study, as m^6^A modification can regulate translation, degradation and other RNA processes. The results of methylated RNA immunoprecipitation (MeRIP) confirmed m^6^A modification of both *STEAP3-AS1* and *STEAP3* mRNA (Fig. 5B). In addition, RNA pulldown and RIP analysis showed that *STEAP3-AS1* could directly bind to YTHDF2 (Fig. 5C-D). To map the binding fragment of *STEAP3-AS1* responsible for YTHDF2 interaction, we generated truncated mutants of *STEAP3-AS1* (1–758 bp (F1), 759–1988 bp (F2) and 1989–3218 bp (F3)) (Fig. 5E). Further RNA pulldown assay revealed that the 3′-fragment (1989–3218 bp, F3) of *STEAP3-AS1* was responsible for its interaction with YTHDF2 (Fig. 5E), which was consistent with the predicted sequence from the AnnoLnc2 database (Fig. S5A).

To determine the related regulators for *STEAP3* m^6^A modification, we knocked down m^6^A writers (*METTL3*, *METTL14*) and m^6^A readers (*YTHDF1*, *YTHDF2*) in CRC cells. As shown in Fig. S5B and Fig. S5C, the protein level and mRNA level of STEAP3 were decreased by METTL14 and YTHDF2, indicating that METTL14-mediated m^6^A modification of STEAP3 might promote its degradation via YTHDF2. Moreover, MeRIP analysis of *STEAP3* in *METTL14*-knockdown CRC cells confirmed that METTL14 is the m^6^A writer for *STEAP3* (Fig. S5D). The putative m^6^A modification sites are in exon 3 and exon 2, respectively, for *STEAP3* and *STEAP3-AS1*, as predicted by SRAMP database (http://www.cuilab.cn/sramp) (Fig. S5E).

As *STEAP3* mRNA underwent m^6^A modification (Fig. 5B) but exhibited no obvious interaction with YTHDF2, we speculated that *STEAP3-AS1* may bind to YTHDF2 thus disrupting *STEAP3*-YTHDF2 interaction. To verify this hypothesis, RNA pulldown and RIP assay were performed in control or *STEAP3-AS1*-knockdown CRC cells. The data revealed that knockdown of *STEAP3-AS1* significantly increased *STEAP3* binding to YTHDF2 (Fig. 5F-G). Furthermore, *STEAP3-AS1*-knockdown resulted in accelerated *STEAP3* degradation as examined by relative mRNA levels after actinomycin D (ACD) treatment (Fig. 5H), while overexpression of *STEAP3-AS1* showed the opposite effect (Fig. 5I). Collectively, these results indicate that *STEAP3-AS1* binds to YTHDF2 to prevent m^6^A-mediated degradation of *STEAP3* mRNA.

### *STEAP3-AS1* activates Wnt/β-catenin signaling to promote CRC progression

**Fig. 6:**
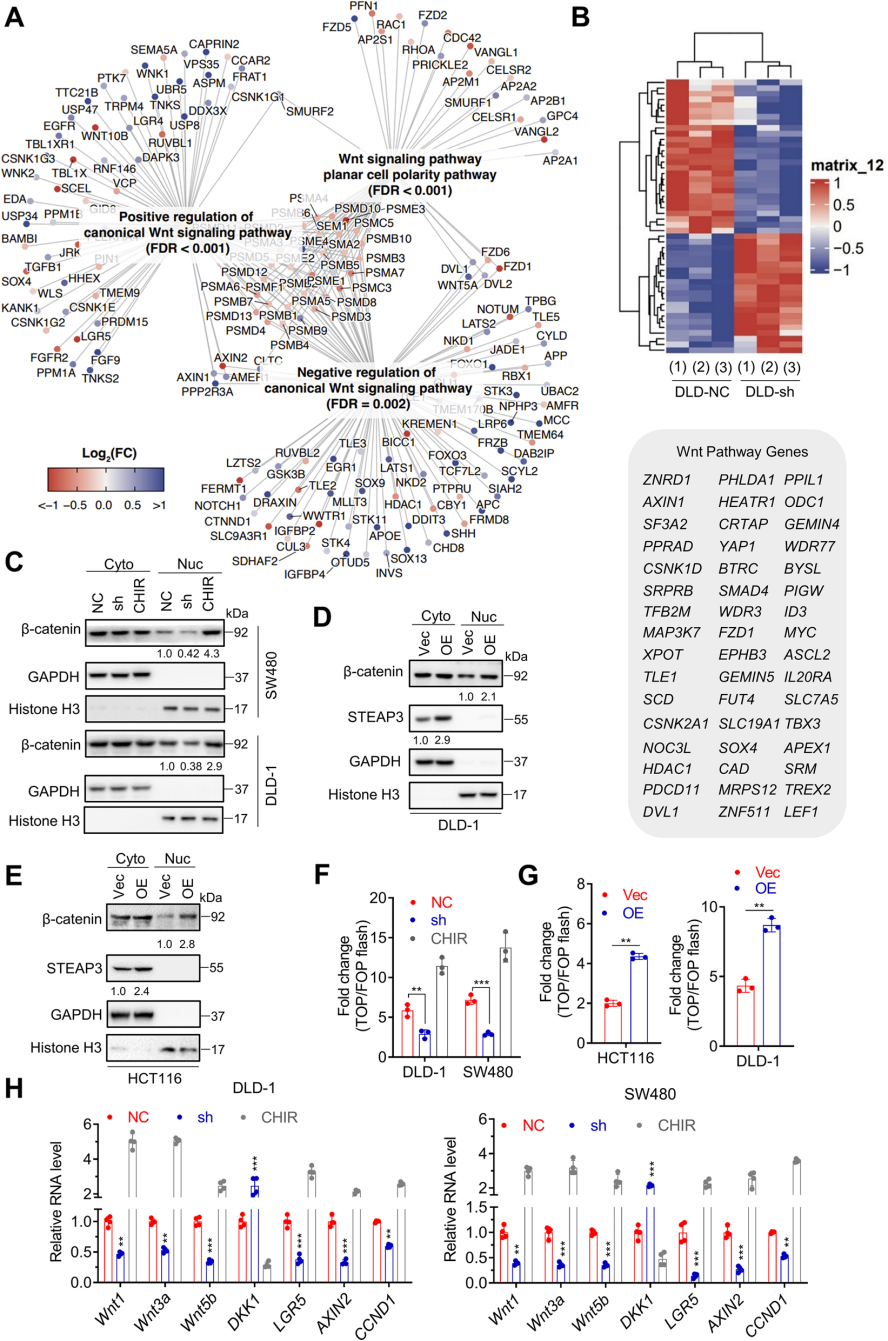
*STEAP3-AS1* activates wnt/β-catenin signaling to promote CRC progression. **A** Gene-Concept Network of enriched Wnt signaling pathways based on RNA-sequencing analysis of control and *STEAP3-AS1*-knockdown DLD-1 cells. **B** Heatmap of RNA sequencing results from Fig. 6**A** showing the expression patterns of Wnt signaling pathway-related genes. **C** Western blotting analysis showing the expression level of cytoplasmic (Cyto) and nuclear (Nuc) β-catenin in DLD-1 and SW480 cells with or without *STEAP3-AS1* knockdown. The GSK3β inhibitor CHIR-99021 was used as a positive control. **D-E** The expression level of cytoplasmic (Cyto) and nuclear (Nuc) β-catenin in control or *STEAP3-AS1*-overexpressing DLD-1 (**D**) and HCT116 (**E**) cells was analyzed by western blotting. **F** TOP/FOP flash reporter assay in control or *STEAP3-AS1*-knockdown DLD-1 and SW480 cells. The GSK3β inhibitor CHIR-99021 was used as a positive control. **G** TOP/FOP flash reporter assay in control or *STEAP3-AS1*-overexpressing HCT116 (left) and DLD-1 (right) cells. **H** Relative RNA levels of several Wnt members and the Wnt inhibitor protein *DKK1* in control or *STEAP3-AS1*-knockdown DLD-1 and SW480 cells. The GSK3β inhibitor CHIR-99021 was used as a positive control. Data are means ± s.d. and are representative of at least 3 independent experiments. (** *P* < 0.01 and *** *P* < 0.001)

To identify the specific signaling pathway responsible for *STEAP3-AS1*-mediated CRC progression, RNA sequencing and the following pathways enrichment analyses were performed using control and *STEAP3-AS1*-knockdown DLD-1 cells. As shown in Fig. 6A, enriched biological pathways analysis revealed Wnt signaling as one of the most significantly changed pathways in *STEAP3-AS1*-knockdown cells, with the expression levels of 48 related genes significantly altered (Fig. 6B).

To validate the effect of *STEAP3-AS1*, activation of the Wnt signaling pathway was firstly examined by nuclear translocation of β-catenin in CRC cells with *STEAP3-AS1* manipulation (the GSK3β inhibitor CHIR-99021 was used as a positive control). As shown in Fig. 6C-E, *STEAP3-AS1* knockdown resulted in a decreased level of nuclear β-catenin, while *STEAP3-AS1* overexpression increased the level of nuclear β-catenin. Consistently, immunofluorescence analysis demonstrated that β-catenin was primarily located in cytoplasm in *STEAP3-AS1*-knockdown CRC cells (Fig. S6A). In addition, the interaction of the β-catenin/Axin/GSK3β complex, which is essential for Wnt signaling inhibition, was impaired in HCT116 cells following *STEAP3-AS1* overexpression (Fig. S6B-C). Furthermore, results of the TOP/FOP flash assay, which reflects the transcriptional activity of β-catenin-TCF, showed that *STEAP3-AS1* knockdown significantly weakened the transcriptional activity of β-catenin-TCF in CRC cells (Fig. 6F), whereas enhanced expression of *STEAP3-AS1* increased the activity (Fig. 6G). Also, relative mRNA levels of several Wnt downstream genes (including *Wnt1*, *Wnt3a*, *Wnt5b*, *LGR5*, *AXIN2*, and *CCND1*) were decreased, while the Wnt inhibitor protein DKK1 was enhanced in *STEAP3-AS1*-knockdown CRC cells (Fig. 6H). The results in *STEAP3-AS1*-overexpressing CRC cells were exactly opposite (Fig. S6D). Altogether, these results illustrate that *STEAP3-AS1* activates Wnt/β-catenin signaling to promote CRC progression. More importantly, *STEAP3-AS1* knockdown-triggered cytoplasmic retention of β-catenin was rescued by STEAP3 reintroduction (Fig. S6A), suggesting that STEAP3 plays an important part in *STEAP3-AS1*/Wnt/β-catenin axis.

### *STEAP3-AS1*/STEAP3-mediated Fe^2+^ generation inactivates GSK3β to stimulate Wnt/β-catenin signaling

**Fig. 7:**
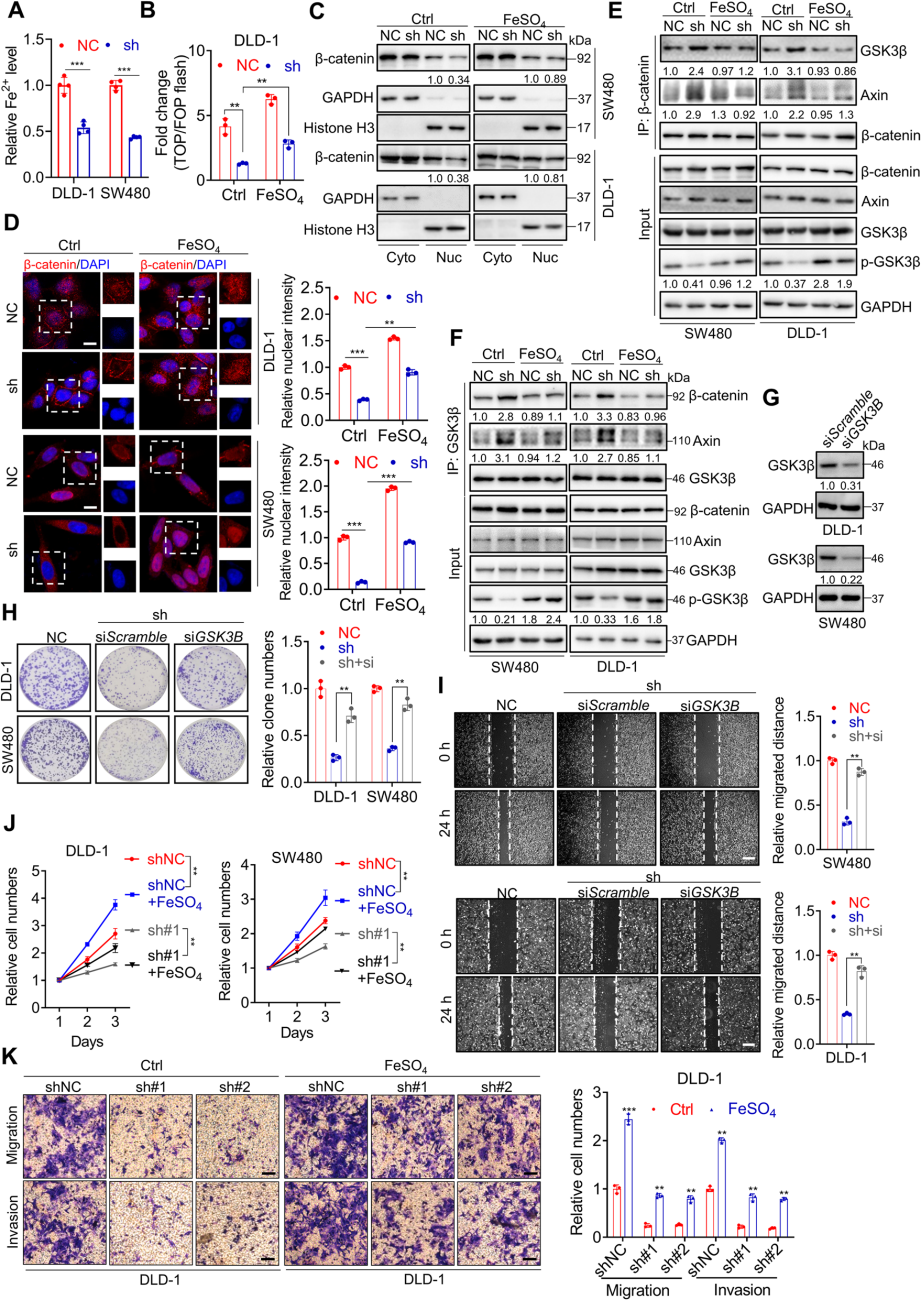
*STEAP3-AS1*/STEAP3-mediated Fe^2+^ inactivates GSK3β to stimulate wnt/β-catenin signaling. **A** Relative cellular Fe^2+^ level in DLD-1 and SW480 cells with or without *STEAP3-AS1* knockdown. **B** TOP/FOP flash assay for detecting the transcriptional activity of Wnt/β-catenin signaling in control or *STEAP3-AS1* knockdown DLD-1 cells with or without FeSO_4_ (100 μM) treatment for 48 h. **C** After treatment with or without FeSO_4_ (100 μM) for 48 h, control or *STEAP3-AS1* knockdown DLD-1 and SW480 cells were analyzed to show the subcellular distribution of β-catenin using nuclear/cytoplasmic fractionation and subsequent WB analysis. (Cyto, cytoplasm; Nuc, nucleus). **D** Control or *STEAP3-AS1* knockdown DLD-1 and SW480 cells were seeded on slides on 24 well-plates overnight and treated as in (**C**), IF assay was conducted to detect the subcellular distribution of β-catenin. Scale bar: 10 μm. (**E**-**F**) Control or *STEAP3-AS1* knockdown DLD-1 and SW480 cells were seeded on a culture dish overnight and treated as in (**C**). Immunoprecipitation (IP) and WB analysis were performed to observe the interaction between β-catenin and GSK3β. **G** The efficiency of siRNA-mediated GSK3β knockdown was measured by western blotting. **H** The colony formation of control and *STEAP3-AS1* knockdown DLD-1 and SW480 cells treated with or without GSK3β siRNA. **I** Wound healing assay and relative migration distance of control and *STEAP3-AS1*-knockdown DLD-1 and SW480 cells treated with or without GSK3β siRNA. Scale bar: 100 μm. **J** Control or *STEAP3-AS1* knockdown DLD-1 and SW480 cells were treated as in (**C**). Cell growth was performed by MTT assay over a 3-day period. (sh#1, sh*STEAP3-AS1* #1). **K** Control or *STEAP3-AS1* knockdown DLD-1 cells were treated as in (**C**). Migration and invasion of DLD-1 cells were evaluated by transwell assays. Scale bar: 100 μm. (sh#1, sh*STEAP3-AS1* #1; sh#2, sh*STEAP3-AS1* #2). Data are means ± s.d. and are representative of at least 3 independent experiments. (** *P* < 0.01 and *** *P* < 0.001)

STEAP3 is a well-documented metalloreductase involved in reducing Fe^3+^ to Fe^2+^, thus playing an essential role in cancer progression [38–40]. Given the role of Fe^2+^ on phosphorylating and inactivating GSK3β [41–44], we therefore speculated that *STEAP3-AS1* activated Wnt/β-catenin signaling might be related to STEAP3-mediated Fe^2+^ generation. We firstly set about investigating the effect of *STEAP3-AS1* on Fe^2+^ levels. Results suggested that *STEAP3-AS1* knockdown distinctly decreased the cellular Fe^2+^ level in DLD-1 and SW480 cells, while *STEAP3-AS1* overexpression elevated cellular Fe^2+^ level in HCT116 cells (Fig. 7A and S7A). Furthermore, the TOP/FOP flash assays suggested that *STEAP3-AS1* knockdown impaired the transcriptional activity of Wnt/β-catenin signaling, while supplementation of exogenous Fe^2+^ could restore the decreased transcriptional activity of Wnt/β-catenin signaling in DLD-1 and SW480 cells (Fig. 7B and S7B). As nuclear localization of β-catenin is the prerequisite for exerting transcriptional function, we investigated the role of Fe^2+^ on the nuclear localization of β-catenin. Nuclear/cytoplasmic fractionation and subsequent WB analysis, together with IF assay revealed that *STEAP3-AS1* knockdown hindered the nuclear localization of β-catenin, while supplementation of exogenous Fe^2+^ could restore its nuclear localization (Fig. 7C-D). As activity of β-catenin was reported to be regulated by GSK3β [44], we conducted experiments to determine whether GSK3β was involved in the effect of Fe^2+^ on β-catenin activity. Co-IP and subsequent WB analysis suggested that *STEAP3-AS1* knockdown decreased Ser 9 phosphorylation of GSK3β (p-GSK3β) with little influence on the total GSK3β protein levels, and *STEAP3-AS1* deficiency significantly enhanced the interaction between β-catenin and GSK3β or Axin (Fig. 7E-F). As expected, addition of Fe^2+^ could diminish the interaction between β-catenin and GSK3β or Axin (Fig. 7E-F). To further ascertain the function of GSK3β in *STEAP3-AS1*-mediated CRC progression, we performed siRNA-mediated knockdown of GSK3β expression under the manipulation of *STEAP3-AS1*. As shown in Fig. 7G-I and Fig. S7C-D, knockdown of GSK3β could partly reverse the decreased cell proliferation and rescue the migratory and invasive ability of *STEAP3-AS1*-knockdown CRC cells, further confirming the regulatory role of GSK3β in *STEAP3-AS1*-mediated CRC progression. In summary, our data reveal that the activation of Wnt/β-catenin signaling relies on *STEAP3-AS1*/STEAP3/Fe^2+^ axis mediated GSK3β inactivation.

Next, we employed multiple assays to ascertain the role of Fe^2+^ in *STEAP3-AS1* mediated CRC progression. Cell viability and colony formation assay suggested that supplementation of exogenous Fe^2+^ could partly counteract the corresponding decrease in proliferation rate induced by *STEAP3-AS1* knockdown (Fig. 7J and S7E-F), indicating that Fe^2+^ participated in *STEAP3-AS1* promoted CRC proliferation. Moreover, wound healing and transwell assays also demonstrated that Fe^2+^ addition rescued the migration and invasion in *STEAP3-AS1* knockdown DLD-1 and SW480 cells (Fig. 7K and S7G-H). Additionally, the epithelial phenotype caused by *STEAP3-AS1* knockdown could be partially reversed by Fe^2+^ treatment, as evidenced by the reduction in epithelial markers and augmentation of mesenchymal markers (Fig. S7I). Collectively, our results suggest that Fe^2+^ participates in *STEAP3-AS1*/STEAP3/Wnt/β-catenin axis mediated CRC proliferation and metastasis.

## Discussion

Numerous studies support the important functions of hypoxia in facilitating tumor progression by regulating abnormal expression of proteins. The role of lncRNAs in hypoxia-mediated tumor progression remains largely elusive. In the present study, we identified that *STEAP3-AS1* is a hypoxia-responsive lncRNA, which was transcribed by HIF-1α through binding to the HREs located near the *STEAP3-AS1* locus. Upregulation of *STEAP3-AS1* promoted proliferation and metastasis of CRC cells both in vitro and in vivo and was positively correlated with poor prognosis of CRC patients. Further studies found that *STEAP3-AS1* conferred the upregulation of STEAP3 protein by interacting with YTHDF2 to prevent m^6^A-mediated degradation of *STEAP3* mRNA, thus preserving Fe^2+^ concentration to activate Wnt/β-catenin and favor CRC progression. To the best of our knowledge, this is the first report identifying *STEAP3-AS1* as a hypoxia-responsive lncRNA in CRC and elucidating the mechanisms underlying *STEAP3-AS1*-mediated CRC progression. These findings indicate that lncRNA *STEAP3-AS1* may serve as a potential biomarker for clinical CRC management.

*STEAP3-AS1* is an antisense lncRNA of six-transmembrane epithelial antigen of the prostate 3 (STEAP3, also known as TSAP6 or dudulin-2), which was initially identified as a potential prognostic biomarker in tongue squamous cell carcinoma (TSCC) [45]. In addition, *STEAP3-AS1* also displayed an oncogenic role in human hepatocellular carcinoma (HCC) by acting as a competing endogenous RNA (ceRNA) and served as a risk scoring system together with three other lncRNAs (SNHG1, RUSC1-AS1, and SNHG3) to predict the outcomes of HCC patients [46]. Recently, *STEAP3-AS1* was also reported to regulate cell cycle by modulating CDKN1C expression in colon cancer, but the detailed mechanisms were not fully understood [47]. In this study, we found that hypoxia-induced upregulation of *STEAP3-AS1* accelerated the proliferation and metastasis of CRC cells both in vitro and in vivo and was positively correlated with the poor outcomes of CRC patients, suggesting its potential application in clinical management of CRC. Moreover, *STEAP3-AS1* caused the expression of its neighboring STEAP3 in a m^6^A modification-dependent manner, which activated Wnt/β-catenin signaling to support CRC progression. Importantly, recent in silico analysis of a serum exosome-derived ceRNA network has identified *STEAP3-AS1* as an independent prognostic predictor of glioblastoma and gallbladder cancer [48, 49], further proving its clinic value as a promising biomarker for cancers. Therefore, further studies elucidating the functions of secreted *STEAP3-AS1* in CRC progression may expand its applications for early detection or prediction of drug response in CRC patients.

Mounting evidence has indicated that antisense transcript lncRNA can positively or negatively regulate the expression of its nearby protein-coding genes [50]. For example, lncRNA *FOXC2-AS1* directly bound to FOXC2 mRNA and increased its expression to confer doxorubicin resistance in osteosarcoma [51], while lncRNA *HOXD-AS1* mediated the recruitment of PRC2 to the *HOXD3* promoter to significantly repress the transcription of the *HOXD3* gene [52]. Indeed, the negative correlation between *STEAP3-AS1* and its neighboring STEAP3 has been previously reported, but the regulatory mechanism was not clear [47]. However, in the present study, we demonstrated that *STEAP3-AS1* interacted with the m^6^A reader YTHDF2 to inhibit m^6^A-mediated degradation of *STEAP3* mRNA and upregulate STEAP3 protein expression to promote CRC progression. Moreover, exogenous overexpression of STEAP3 could partially rescue *STEAP3-AS1* knockdown-mediated inhibition of CRC progression, implying a positive correlation between *STEAP3-AS1* and STEAP3. As STEAP3 is a metalloreductase responsible for reducing cellular Fe^3+^ to Fe^2+^ [53], increased expression of STEAP3 preserved cellular Fe^2+^ concentrations to phosphorylate and inactivate GSK3β, thus activating Wnt/β-catenin signaling to favor CRC progression. In fact, the oncogenic roles of STEAP3 have already been found in HCC and glioblastoma [40, 54], which could partially strengthen our conclusion that *STEAP3-AS1* positively correlated with STEAP3 to promote CRC progression.

m^6^A is one of the most prevalent RNA modifications, which regulates RNA splicing, translation, export, and stability, especially within lncRNAs and mRNAs [55]. Increasing studies have reported that aberrant regulations of m^6^A modification on certain RNAs executed important functions to modulate tumor initiation and progression [56, 57]. Moreover, proteins responsible for m^6^A modification, including writers (such as METTL3/14), erasers (such as FTO and ALKBH5), and readers (such as YTHDF1/2/3), have been found to be overexpressed and promote the initiation and development of many cancer types [58–60]. Accumulating data suggest that YTHDF2, a reader protein responsible for m^6^A-mediated mRNA decay, is closely related to many aspects of human cancers by regulating multiple biological processes, such as metastasis, proliferation, differentiation and inflammation [61–63]. In our study, the m^6^A modification was found to occur both on lncRNA *STEAP3-AS1* and *STEAP3* mRNA. Further studies demonstrated that *STEAP3-AS1* bound to YTHDF2 through its 3′-fragment, which prevented m^6^A-mediated degradation of *STEAP3* mRNA, resulting in upregulation of STEAP3 protein expression. Further investigations are required to clarify the binding motif of YTHDF2 with lncRNA *STEAP3-AS1*.

The reprogramming of iron metabolism is an indispensable biological process for cancer cells, which contributes to the initiation, growth, and metastasis of tumors [64]. The STEAP3-mediated reduction of ferric iron (Fe^3+^) to ferrous iron (Fe^2+^) is the major aspect of iron utilization in cancer cells [65, 66]. A growing body of evidence has revealed that sufficient cellular Fe^2+^ is essential for the activation of Wnt signaling, particularly in CRC cells, during which the regulation of β-catenin is characterized as a key event. However, to date, the mechanisms are not clearly defined [67, 68]. In this study, by performing RNA-seq analysis and assessing Wnt activation, we confirmed the important functions of Wnt/β-catenin signaling in *STEAP3-AS1*-mediated CRC progression. We also found that exogenous supplementation of Fe^2+^ could partially reverse the inhibitory effect on Wnt/β-catenin signaling and CRC progression caused by *STEAP3-AS1* knockdown, indicating the pivotal role of *STEAP3-AS1*/STEAP3-mediated Fe^2+^ generation in activating Wnt and promoting CRC progression. Moreover, an abundant supply of Fe^2+^ increased the Ser 9 phosphorylation of GSK3β and inhibited its kinase activity, thus releasing β-catenin for nuclear translocation to activate Wnt signaling. This observation is consistent with the previous findings that Fe^2+^ could promote the phosphorylation of GSK3β on Ser 9 in hippocampal neurons and this phosphorylation of GSK3β inhibited its activity and activated Wnt/β-catenin signaling in cancer cells [42, 44]. However, further studies are required to elucidate the mechanisms underlying Fe^2+^-mediated Ser 9 phosphorylation and inactivation of GSK3β in cancer cells.

## Conclusions

In summary, we identified a novel hypoxia-induced lncRNA *STEAP3-AS1* and elucidated its regulatory mechanisms in facilitating CRC progression. *STEAP3-AS1* conferred the expression of STEAP3 by interacting with YTHDF2 to prevent m^6^A-mediated degradation of STEAP3 mRNA. Enhanced STEAP3 expression promoted cellular Fe^2+^ concentration and induced the Ser 9 phosphorylation of GSK3β, thus activating Wnt/β-catenin signaling to accelerate CRC progression. These findings have revealed the mechanisms of *STEAP3-AS1*-induced CRC progression by regulating the STEAP3/GSK3β/Wnt/β-catenin axis, which may act as a possible biomarker or target for the diagnosis and treatment of CRC.

## Supplementary Information

**Additional file 1: Fig. S1.** LncRNA *STEAP3-AS1* is upregulated under hypoxia in CRC. (A) The correlation between the expression of *STEAP3-AS1* and hypoxia signature genes in TCGA datasets was analyzed by Pearson correlation test. (B-F) *STEAP3-AS1* expression in marginal and inner regions from DLD-1 xenografts was analyzed by qPCR assay. (G) Immunohistochemical staining was performed to determine the levels of HIF-1α in marginal and inner regions from DLD-1 xenografts. In situ hybrization was performed to determine the levels of *STEAP3-AS1* in marginal and inner regions from DLD-1 xenografts. Scale bar: left 50 μm, right 20 μm. (H) Schematic diagram of HREs in *STEAP3-AS1* genome. (I) The coding potential of *STEAP3-AS1* was predicted by CPC2 and CPAT database. (J) The expression level of lncRNA *STEAP3-AS1* in the subcellular fractions of SW480, RKO and LoVo ells was detected by qRT-PCR. U6 and GAPDH were used as nuclear and cytoplasmic markers, respectively. Data are means ± s.d. and are representative of at least 3 independent experiments. (** *P* < 0.01 and *** *P* < 0.001).

**Additional file 2: Fig. S2.** LncRNA *STEAP3-AS1* increases the proliferation rate of CRC cells. (A) Colony formation assays were conducted to determine the effects of lncRNA *STEAP3-AS1* knockdown on the proliferation of HT29 and LoVo cells. (sh#1, sh*STEAP3-AS1* #1; sh#2, sh*STEAP3-AS1* #2). (B) The statistic graph of relative clone numbers in (A). (sh#1, sh*STEAP3-AS1* #1; sh#2, sh*STEAP3-AS1* #2). (C) Relative *STEAP3-AS1* expression was detected using qPCR analysis in HCT116 cells with or without *STEAP3-AS1* overexpression. (OE, *STEAP3-AS1* overexpression). (D-E) The effects of lncRNA *STEAP3-AS1* overexpression on the proliferation of HCT116 cells were examined by MTT assays (D) and colony formation assays (E). (OE, *STEAP3-AS1* overexpression). (F) EdU assay was conducted to determine the proliferation rate of HCT116 cells with or without *STEAP3-AS1* overexpression. Scale bar: 50 μm. (OE, *STEAP3-AS1* overexpression). Data are means ± s.d. and are representative of at least 3 independent experiments. (** *P* < 0.01 and *** *P* < 0.001).

**Additional file 3: Fig. S3.** LncRNA *STEAP3-AS1* regulates migration and invasion of CRC cells. (A) Number of lung metastatic nodules in DLD-1 cells tail vein injection models. (B) Schematic diagram describing the establishment of splenic injection and orthotopic implantation models. (C) Wound healing assay showing cell migration of vector or *STEAP3-AS1*-overexpressing HCT116 cells. Scale bar: 100 μm. (OE, *STEAP3-AS1* overexpression). (D) Transwell assays showing migration and invasion of vector or *STEAP3-AS1*-overexpressing HCT116 cells. Scale bar: 100 μm. (OE, *STEAP3-AS1* overexpression). (E) EMT markers were detected by WB in HCT116 cells with or without *STEAP3-AS1* overexpression. (OE, *STEAP3-AS1* overexpression). (F) IF assay demonstrating the level of ECAD in DLD-1 cells with or without *STEAP3-AS1* knockdown. Scale bar: 10 μm. (sh#1, sh*STEAP3-AS1* #1; sh#2, sh*STEAP3-AS1* #2). Data are means ± s.d. and are representative of at least 3 independent experiments. (*** *P* < 0.001).

**Additional file 4: Fig. S4.** LncRNA *STEAP3-AS1* positively correlates with STEAP3 to promote CRC progression. (A-B) The protein levels of STEAP3 and HIF-1α, and relative *STEAP3* mRNA level in DLD-1 and SW480 cells treated with DMOG (1 mM) or CoCl_2_ (100 μM) were determined by WB and qPCR. (C-D) The protein levels (C) and relative RNA levels (D) of STEAP3 and HIF-1α in the marginal region or the inner region of tumors from Fig. S1A. (E-F) Relative cell numbers at serial time points (E), colony formation (F) and relative clone numbers of control and *STEAP3-AS1*-knockdown SW480 cells with or without replenishment of STEAP3. (sh#1, sh*STEAP3-AS1* #1; sh#2, sh*STEAP3-AS1* #2). (G-H) Wound healing assay (H) and relative migration distance (G) of control and *STEAP3-AS1*-knockdown SW480 cells with or without reintroduction of STEAP3. Scale bar: 100 μm. (sh#1, sh*STEAP3-AS1* #1; sh#2, sh*STEAP3-AS1* #2). (I) Migration and invasion of control and *STEAP3-AS1*-knockdown SW480 cells with or without reintroduction of STEAP3. Scale bar: 100 μm. (sh#1, sh*STEAP3-AS1* #1; sh#2, sh*STEAP3-AS1* #2). (J-K) Migration and invasion of control and *STEAP3-AS1*-overexpressing DLD-1 (J) and SW480 (K) cells treated with or without STEAP3 siRNA. Scale bar: 100 μm. (OE, *STEAP3-AS1* overexpression). Data are means ± s.d. and are representative of at least 3 independent experiments. (** *P* < 0.01 and *** *P* < 0.001).

**Additional file 5: Fig. S5.** *STEAP3-AS1* and *STEAP3* mRNA undergo m^6^A modification in CRC cells. (A) The binding region of *STEAP3-AS1* to YTHDF2 predicted by AnnoLnc2 database. (B) WB analysis of the STEAP3 protein levels in DLD-1 and SW480 cells transfected with si*METTL3*, si*METTL14*, si*YTHDF1* or si*YTHDF2*. (C) Relative RNA levels of *STEAP3-AS1* and *STEAP3* mRNA in DLD-1 and SW480 cells transfected with si*METTL3*, si*METTL14*, si*YTHDF1* or si*YTHDF2*. (D) MeRIP assay showing m^6^A modification of *STEAP3* mRNA in DLD-1 and SW480 cells transfected with si*Scramble* or si*METTL14*. (E) Bioinformatic prediction of m^6^A modification sites in *STEAP3* mRNA and *STEAP3-AS1* using SRAMP database. Data are means ± s.d. and are representative of at least 3 independent experiments. (*** *P* < 0.001).

**Additional file 6: Fig. S6.** *STEAP3-AS1* positively regulates wnt/β-catenin signaling in CRC cells. (A) Immunofluorescence staining of β-catenin in control or *STEAP3-AS1*-knockdown DLD-1 and SW480 cells with or without reintroduction of STEAP3. Scale bar: 10 μm. (B-C) Co-immunoprecipitation analysis of the interaction between β-catenin (B) or GSK3β (C) and their cofactors in HCT116 cells with or without *STEAP3-AS1* overexpression. (D) Relative RNA levels of several Wnt members in HCT116 and DLD-1 cells with or without *STEAP3-AS1* overexpression. Data are means ± s.d. and are representative of at least 3 independent experiments. (** *P* < 0.01 and *** *P* < 0.001).

**Additional file 7: Fig. S7.** Fe^2+^ is essential for *STEAP3-AS1*-mediated CRC progression. (A) Relative cellular Fe^2+^ level in vector or *STEAP3-AS1*-overexpressing HCT116 cells. (OE, *STEAP3-AS1* overexpression). (B) TOP/FOP flash assay for detecting the transcriptional activity of Wnt/β-catenin signaling in control or *STEAP3-AS1* knockdown SW480 cells with or without FeSO_4_ (100 μM) treatment for 48 h. (C-D) Migration and invasion of control and *STEAP3-AS1* knockdown DLD-1 (left) and SW480 (right) cells treated with or without GSK3β siRNA. Scale bar: 100 μm. (E-F) Colony formation assay showing the proliferation rate of control or *STEAP3-AS1* knockdown DLD-1 and SW480 cells with or without FeSO_4_ (100 μM) treatment for 48 h. (G) Would healing assay showing the migration of control or *STEAP3-AS1* knockdown DLD-1 and SW480 cells with or without FeSO_4_ (100 μM) treatment for 48 h. Scale bar: 100 μm. (sh#1, sh*STEAP3-AS1* #1; sh#2, sh*STEAP3-AS1* #2). (H) Transwell assays showing the migration and invasion of control or *STEAP3-AS1* knockdown SW480 cells with or without the treatment of FeSO_4_ (100 μM) for 48 h. Scale bar: 100 μm. (sh#1, sh*STEAP3-AS1* #1; sh#2, sh*STEAP3-AS1* #2). (I) Western blot showing the expression of EMT markers in control or *STEAP3-AS1* knockdown DLD-1 and SW480 cells with or without FeSO_4_ (100 μM) treatment for 48 h. Data are means ± s.d. and are representative of at least 3 independent experiments. (** *P* < 0.01 and *** *P* < 0.001).

**Additional file 8: Supplementary Table S1.** Sequences of primers used in RT-qPCR assay.

**Additional file 9: Supplementary Table S2.** Sequences of primers used in ChIP-qPCR assay.

## Data Availability

The data that support the findings of this study are available from the corresponding author (hcanhua@hotmail.com) upon reasonable request. The raw sequencing data from this study have been deposited in the SRA database (https://www.ncbi.nlm.nih.gov/sra) with the accession numbers PRJNA861928.

## Abbreviations

ACD: Actinomycin D
CCLE: Cancer cell line encyclopedia
ceRNA: Competing endogenous RNA
ChIP: Chromatin immunoprecipitation
Co-IP: Co-immunoprecipitation
CPAT: Coding potential assessment tool
CPC: Coding potential calculator
CRC: Colorectal cancer
EMT: Epithelial-to-mesenchymal transition
FISH: Fluorescence in situ hybridization
GSK3β: Glycogen synthase kinase 3 beta
H&E: Hematoxylin and eosin
HCC: Hepatocellular carcinoma
HIF-1α: Hypoxia-inducible factor-1α
HOTAIR: HOX transcript antisense RNA
HREs: Hypoxia response elements
IF: Immunofluorescence
IHC: Immunohistochemistry
LncRNAs: Long non-coding RNAs
ISH: In situ hybridization
m^6^A: N^6^-methyladenosine
MeRIP: Methylated RNA immunoprecipitation
STEAP3: Six-transmembrane epithelial antigen of the prostate 3
TSCC: Tongue squamous cell carcinoma
YTHDF2: YTH domain-containing family protein 2
