# Supplementary figures and images for: Hypoxia-induced lncRNA STEAP3-AS1 activates Wnt/β-catenin signaling to promote colorectal cancer progression by preventing m6A-mediated degradation of STEAP3 mRNA
Source: Mol Cancer. 2022 Aug 19;21:168. doi: 10.1186/s12943-022-01638-1 (PMC9392287; doi:10.1186/s12943-022-01638-1)

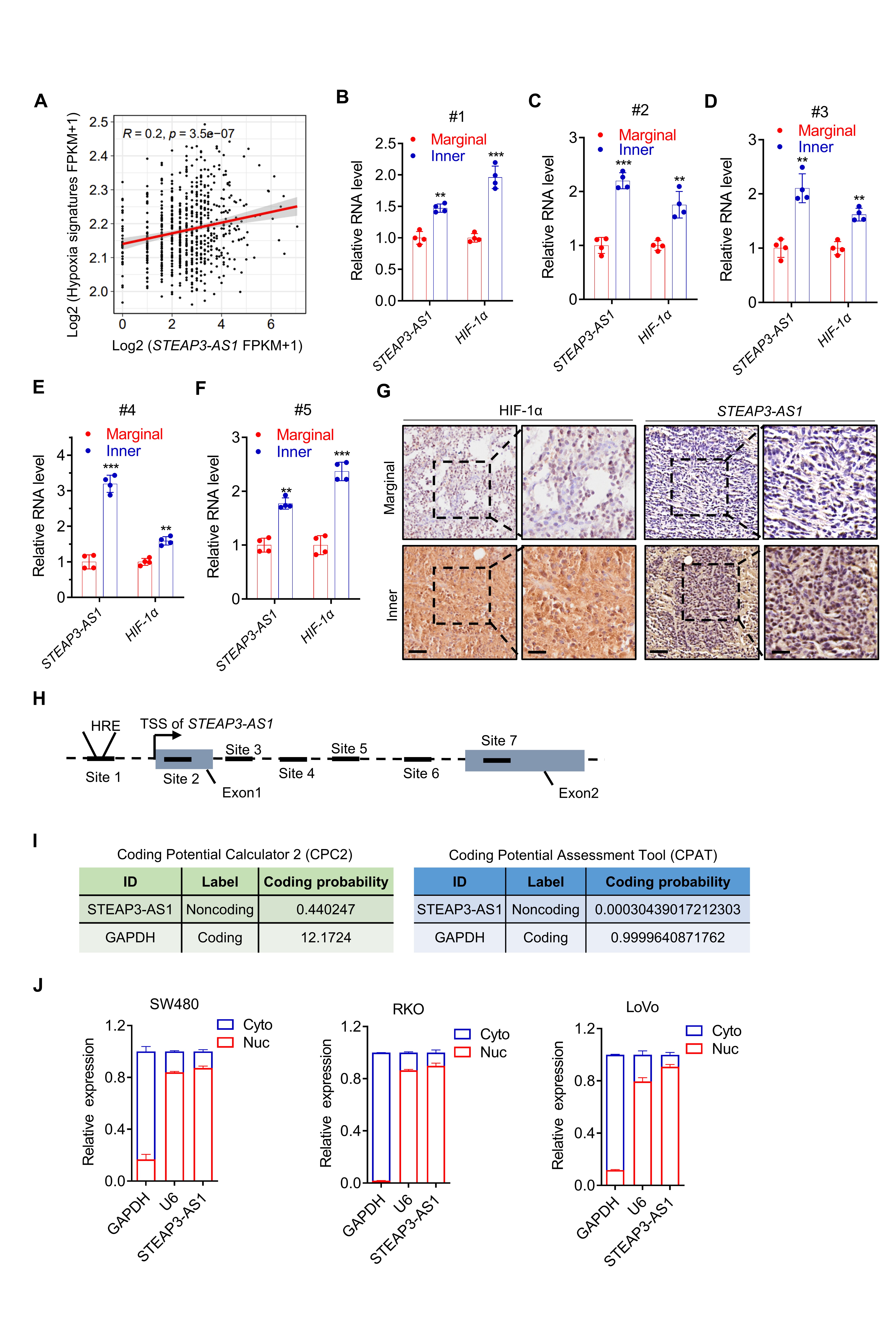

Supplement: Supplementary file 1 — Additional file 1: Fig. S1. LncRNA STEAP3-AS1 is upregulated under hypoxia in CRC. (A) The correlation between the expression of STEAP3-AS1 and hypoxia signature genes in TCGA datasets was analyzed by Pearson correlation test. (B-F) STEAP3-AS1 expression in marginal and inner regions from DLD-1 xenografts was analyzed by qPCR assay. (G) Immunohistochemical staining was performed to determine the levels of HIF-1α in marginal and inner regions from DLD-1 xenografts. In situ hybrization was performed to determine the levels of STEAP3-AS1 in marginal and inner regions from DLD-1 xenografts. Scale bar: left 50 μm, right 20 μm. (H) Schematic diagram of HREs in STEAP3-AS1 genome. (I) The coding potential of STEAP3-AS1 was predicted by CPC2 and CPAT database. (J) The expression level of lncRNA STEAP3-AS1 in the subcellular fractions of SW480, RKO and LoVo ells was detected by qRT-PCR. U6 and GAPDH were used as nuclear and cytoplasmic markers, respectively. Data are means ± s.d. and are representative of at least 3 independent experiments. (** P < 0.01 and *** P < 0.001). [file 12943_2022_1638_MOESM1_ESM.jpg]

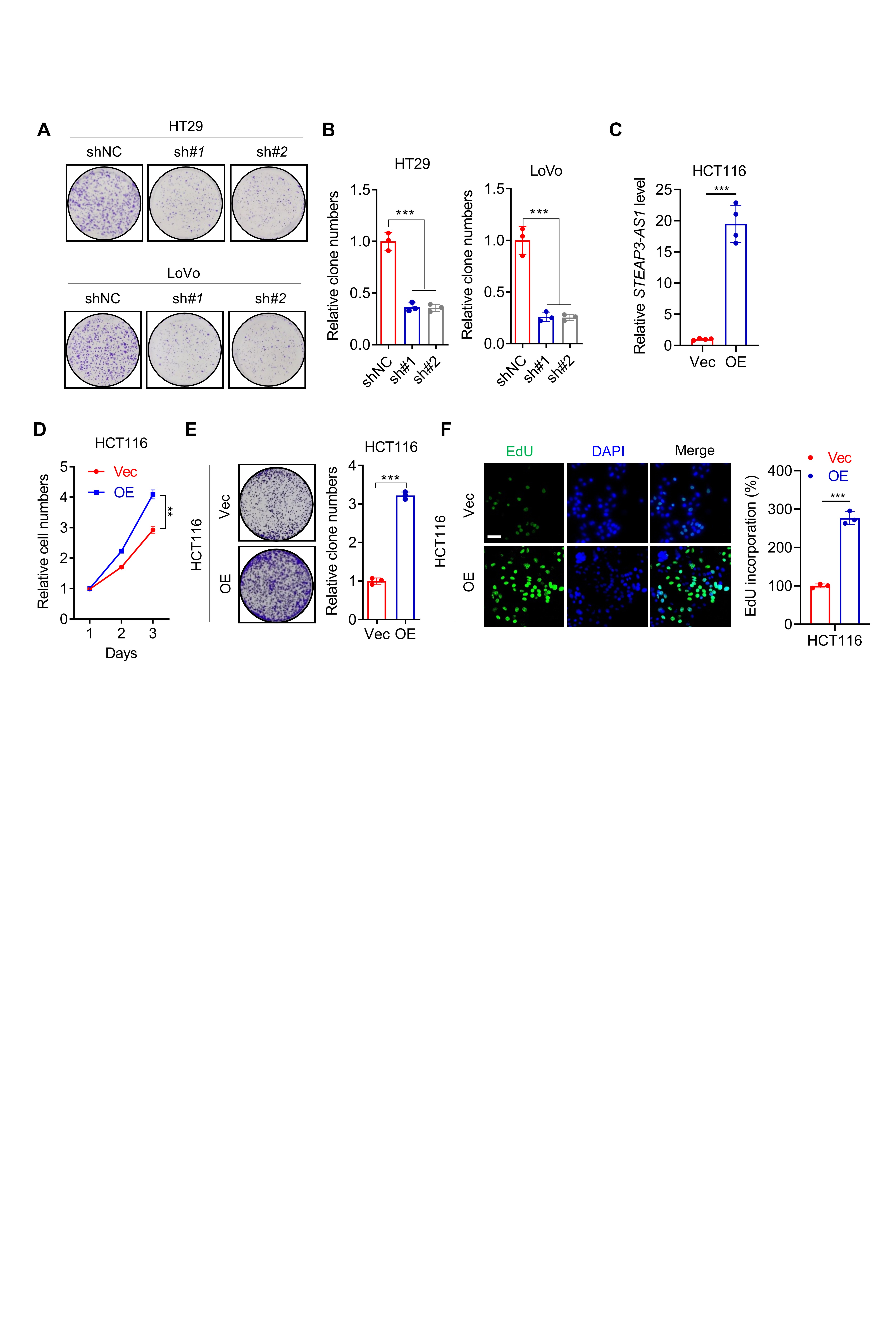

Supplement: Supplementary file 2 — Additional file 2: Fig. S2. LncRNA STEAP3-AS1 increases the proliferation rate of CRC cells. (A) Colony formation assays were conducted to determine the effects of lncRNA STEAP3-AS1 knockdown on the proliferation of HT29 and LoVo cells. (sh#1, shSTEAP3-AS1 #1; sh#2, shSTEAP3-AS1 #2). (B) The statistic graph of relative clone numbers in (A). (sh#1, shSTEAP3-AS1 #1; sh#2, shSTEAP3-AS1 #2). (C) Relative STEAP3-AS1 expression was detected using qPCR analysis in HCT116 cells with or without STEAP3-AS1 overexpression. (OE, STEAP3-AS1 overexpression). (D-E) The effects of lncRNA STEAP3-AS1 overexpression on the proliferation of HCT116 cells were examined by MTT assays (D) and colony formation assays (E). (OE, STEAP3-AS1 overexpression). (F) EdU assay was conducted to determine the proliferation rate of HCT116 cells with or without STEAP3-AS1 overexpression. Scale bar: 50 μm. (OE, STEAP3-AS1 overexpression). Data are means ± s.d. and are representative of at least 3 independent experiments. (** P < 0.01 and *** P < 0.001). [file 12943_2022_1638_MOESM2_ESM.jpg]

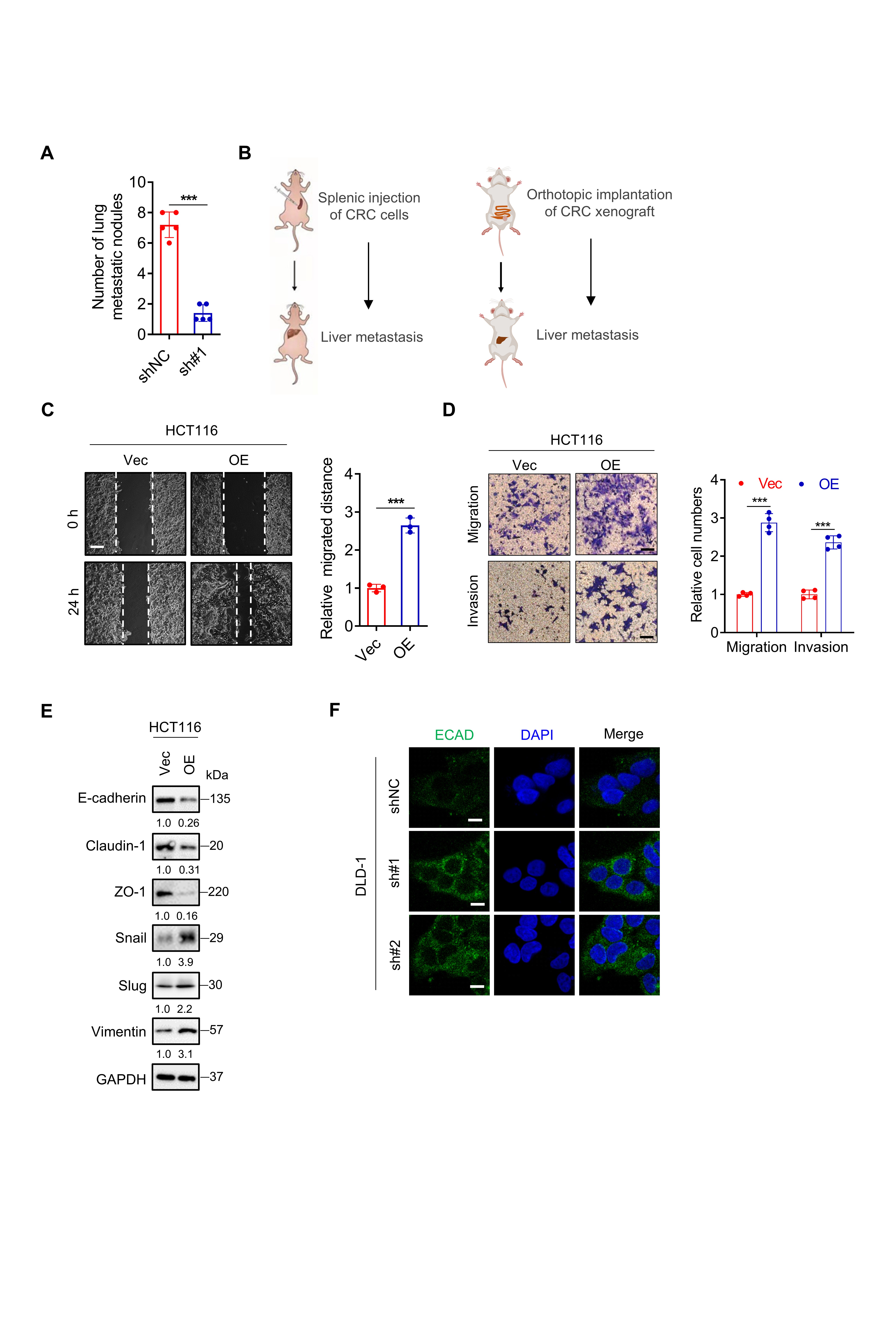

Supplement: Supplementary file 3 — Additional file 3: Fig. S3. LncRNA STEAP3-AS1 regulates migration and invasion of CRC cells. (A) Number of lung metastatic nodules in DLD-1 cells tail vein injection models. (B) Schematic diagram describing the establishment of splenic injection and orthotopic implantation models. (C) Wound healing assay showing cell migration of vector or STEAP3-AS1-overexpressing HCT116 cells. Scale bar: 100 μm. (OE, STEAP3-AS1 overexpression). (D) Transwell assays showing migration and invasion of vector or STEAP3-AS1-overexpressing HCT116 cells. Scale bar: 100 μm. (OE, STEAP3-AS1 overexpression). (E) EMT markers were detected by WB in HCT116 cells with or without STEAP3-AS1 overexpression. (OE, STEAP3-AS1 overexpression). (F) IF assay demonstrating the level of ECAD in DLD-1 cells with or without STEAP3-AS1 knockdown. Scale bar: 10 μm. (sh#1, shSTEAP3-AS1 #1; sh#2, shSTEAP3-AS1 #2). Data are means ± s.d. and are representative of at least 3 independent experiments. (*** P < 0.001). [file 12943_2022_1638_MOESM3_ESM.jpg]

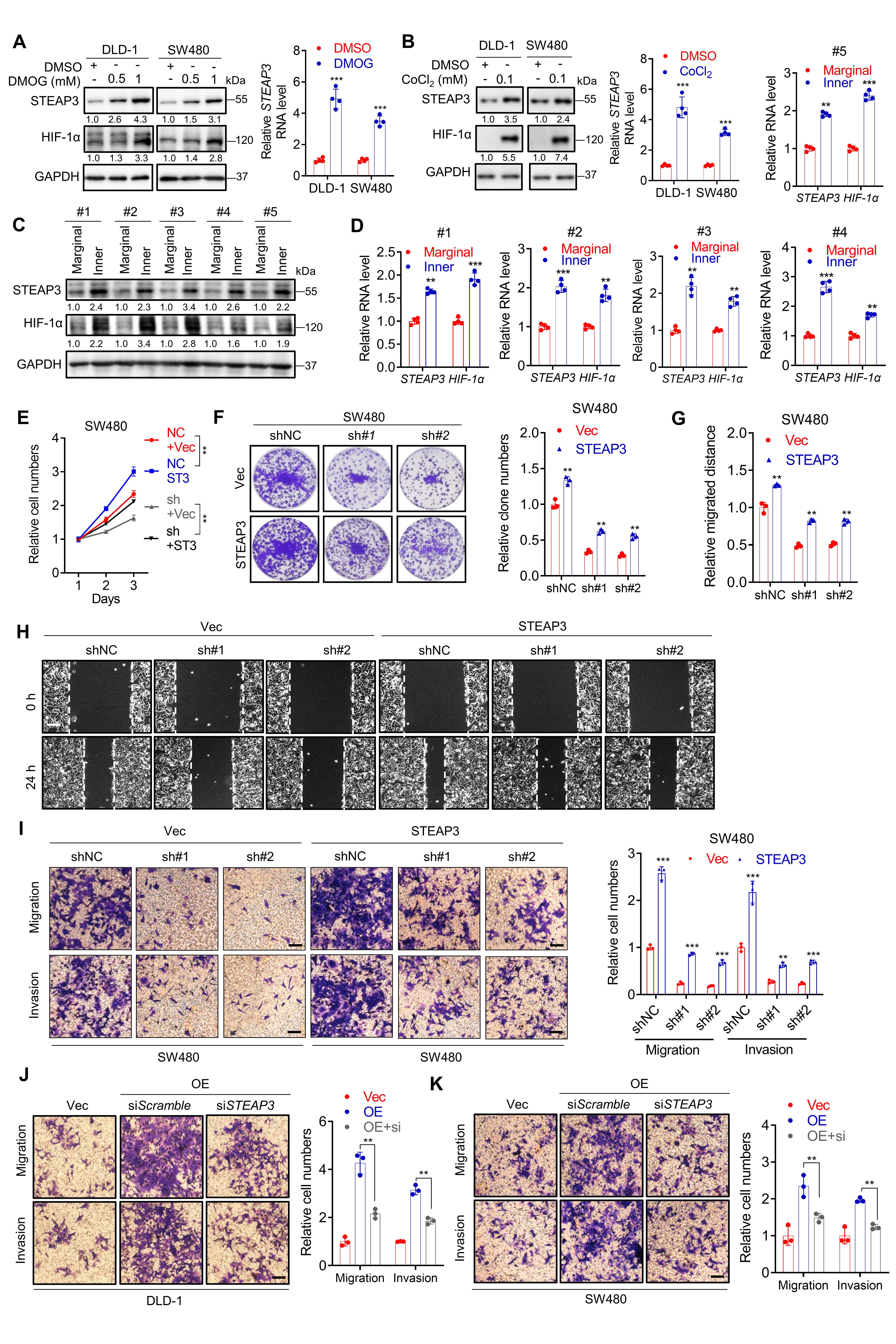

Supplement: Supplementary file 4 — Additional file 4: Fig. S4. LncRNA STEAP3-AS1 positively correlates with STEAP3 to promote CRC progression. (A-B) The protein levels of STEAP3 and HIF-1α, and relative STEAP3 mRNA level in DLD-1 and SW480 cells treated with DMOG (1 mM) or CoCl2 (100 μM) were determined by WB and qPCR. (C-D) The protein levels (C) and relative RNA levels (D) of STEAP3 and HIF-1α in the marginal region or the inner region of tumors from Fig. S1A. (E-F) Relative cell numbers at serial time points (E), colony formation (F) and relative clone numbers of control and STEAP3-AS1-knockdown SW480 cells with or without replenishment of STEAP3. (sh#1, shSTEAP3-AS1 #1; sh#2, shSTEAP3-AS1 #2). (G-H) Wound healing assay (H) and relative migration distance (G) of control and STEAP3-AS1-knockdown SW480 cells with or without reintroduction of STEAP3. Scale bar: 100 μm. (sh#1, shSTEAP3-AS1 #1; sh#2, shSTEAP3-AS1 #2). (I) Migration and invasion of control and STEAP3-AS1-knockdown SW480 cells with or without reintroduction of STEAP3. Scale bar: 100 μm. (sh#1, shSTEAP3-AS1 #1; sh#2, shSTEAP3-AS1 #2). (J-K) Migration and invasion of control and STEAP3-AS1-overexpressing DLD-1 (J) and SW480 (K) cells treated with or without STEAP3 siRNA. Scale bar: 100 μm. (OE, STEAP3-AS1 overexpression). Data are means ± s.d. and are representative of at least 3 independent experiments. (** P < 0.01 and *** P < 0.001). [file 12943_2022_1638_MOESM4_ESM.jpg]

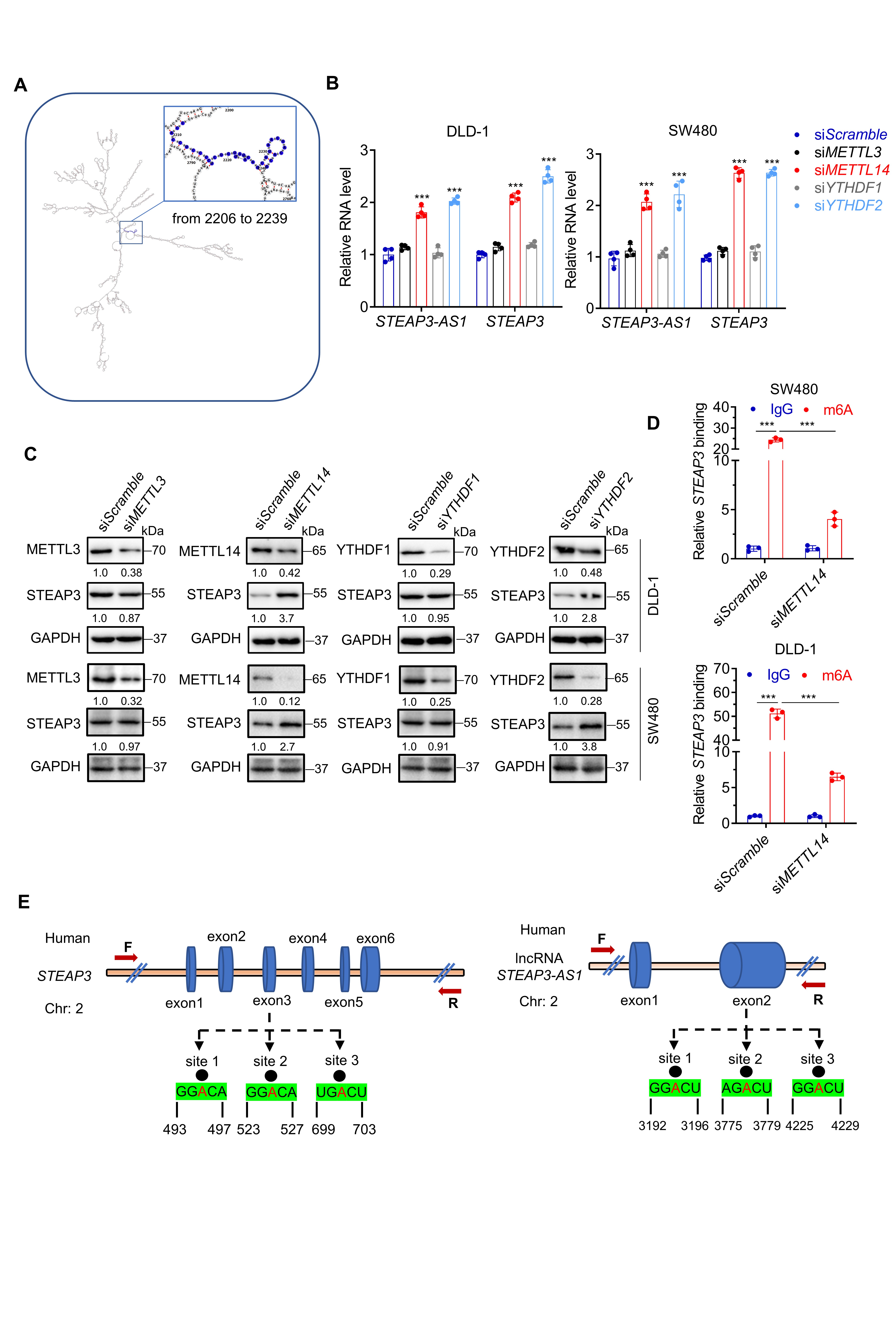

Supplement: Supplementary file 5 — Additional file 5: Fig. S5. STEAP3-AS1 and STEAP3 mRNA undergo m6A modification in CRC cells. (A) The binding region of STEAP3-AS1 to YTHDF2 predicted by AnnoLnc2 database. (B) WB analysis of the STEAP3 protein levels in DLD-1 and SW480 cells transfected with siMETTL3, siMETTL14, siYTHDF1 or siYTHDF2. (C) Relative RNA levels of STEAP3-AS1 and STEAP3 mRNA in DLD-1 and SW480 cells transfected with siMETTL3, siMETTL14, siYTHDF1 or siYTHDF2. (D) MeRIP assay showing m6A modification of STEAP3 mRNA in DLD-1 and SW480 cells transfected with siScramble or siMETTL14. (E) Bioinformatic prediction of m6A modification sites in STEAP3 mRNA and STEAP3-AS1 using SRAMP database. Data are means ± s.d. and are representative of at least 3 independent experiments. (*** P < 0.001). [file 12943_2022_1638_MOESM5_ESM.jpg]

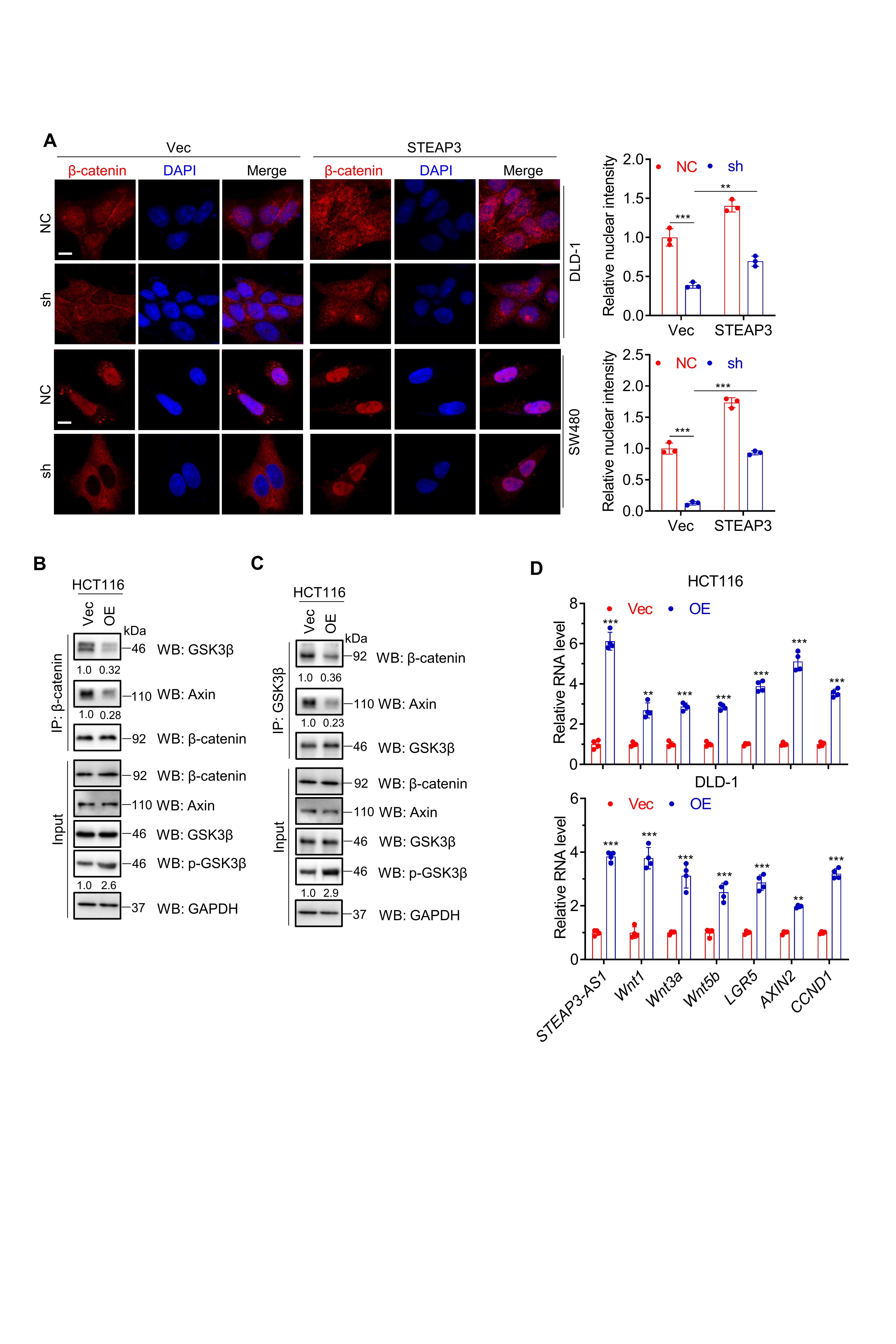

Supplement: Supplementary file 6 — Additional file 6: Fig. S6. STEAP3-AS1 positively regulates wnt/β-catenin signaling in CRC cells. (A) Immunofluorescence staining of β-catenin in control or STEAP3-AS1-knockdown DLD-1 and SW480 cells with or without reintroduction of STEAP3. Scale bar: 10 μm. (B-C) Co-immunoprecipitation analysis of the interaction between β-catenin (B) or GSK3β (C) and their cofactors in HCT116 cells with or without STEAP3-AS1 overexpression. (D) Relative RNA levels of several Wnt members in HCT116 and DLD-1 cells with or without STEAP3-AS1 overexpression. Data are means ± s.d. and are representative of at least 3 independent experiments. (** P < 0.01 and *** P < 0.001). [file 12943_2022_1638_MOESM6_ESM.jpg]

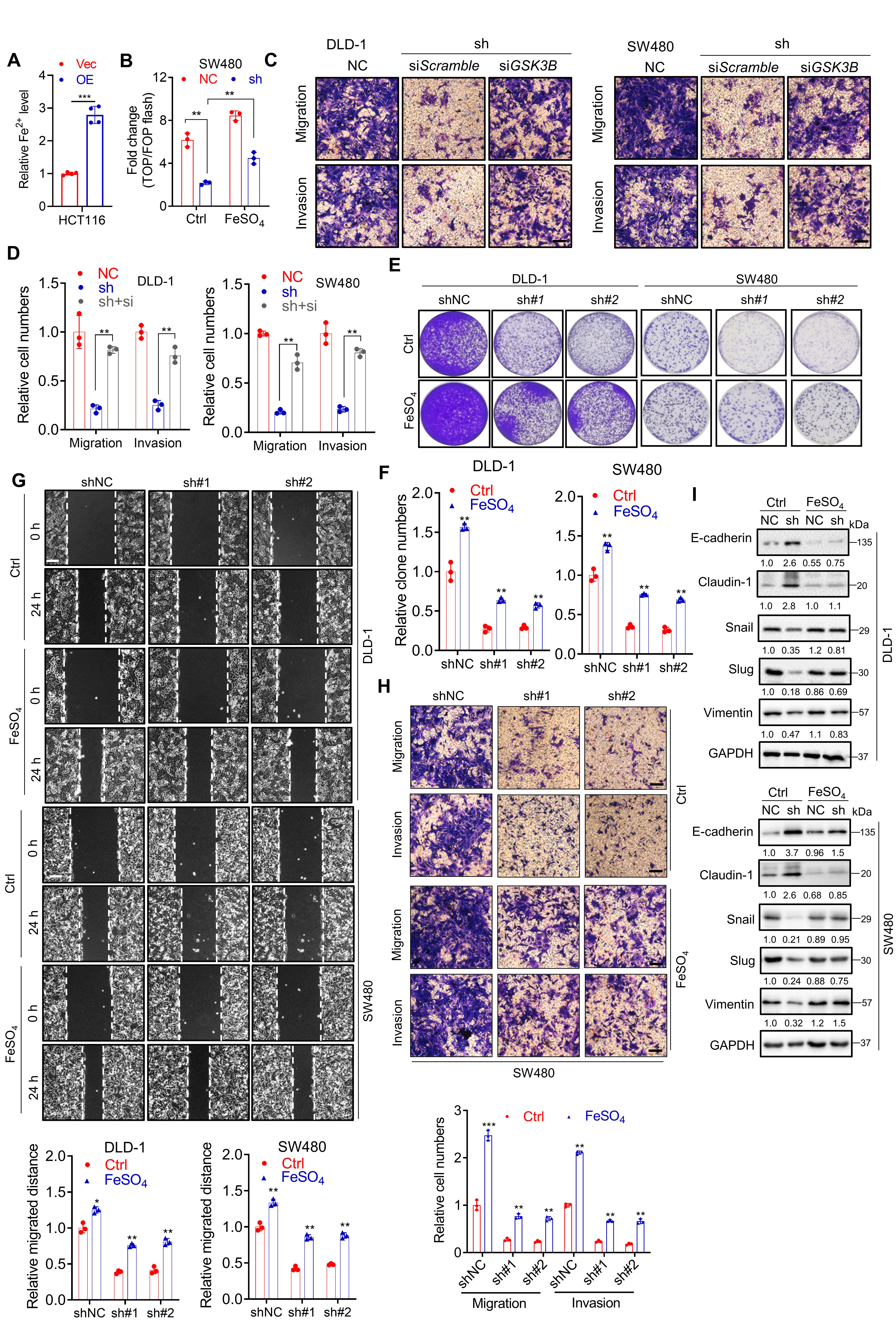

Supplement: Supplementary file 7 — Additional file 7: Fig. S7. Fe2+ is essential for STEAP3-AS1-mediated CRC progression. (A) Relative cellular Fe2+ level in vector or STEAP3-AS1-overexpressing HCT116 cells. (OE, STEAP3-AS1 overexpression). (B) TOP/FOP flash assay for detecting the transcriptional activity of Wnt/β-catenin signaling in control or STEAP3-AS1 knockdown SW480 cells with or without FeSO4 (100 μM) treatment for 48 h. (C-D) Migration and invasion of control and STEAP3-AS1 knockdown DLD-1 (left) and SW480 (right) cells treated with or without GSK3β siRNA. Scale bar: 100 μm. (E-F) Colony formation assay showing the proliferation rate of control or STEAP3-AS1 knockdown DLD-1 and SW480 cells with or without FeSO4 (100 μM) treatment for 48 h. (G) Would healing assay showing the migration of control or STEAP3-AS1 knockdown DLD-1 and SW480 cells with or without FeSO4 (100 μM) treatment for 48 h. Scale bar: 100 μm. (sh#1, shSTEAP3-AS1 #1; sh#2, shSTEAP3-AS1 #2). (H) Transwell assays showing the migration and invasion of control or STEAP3-AS1 knockdown SW480 cells with or without the treatment of FeSO4 (100 μM) for 48 h. Scale bar: 100 μm. (sh#1, shSTEAP3-AS1 #1; sh#2, shSTEAP3-AS1 #2). (I) Western blot showing the expression of EMT markers in control or STEAP3-AS1 knockdown DLD-1 and SW480 cells with or without FeSO4 (100 μM) treatment for 48 h. Data are means ± s.d. and are representative of at least 3 independent experiments. (** P < 0.01 and *** P < 0.001). [file 12943_2022_1638_MOESM7_ESM.jpg]
