# Supplementary material for: Hypoxia-induced lncRNA STEAP3-AS1 activates Wnt/β-catenin signaling to promote colorectal cancer progression by preventing m6A-mediated degradation of STEAP3 mRNA
Source: Mol Cancer. 2022 Aug 19;21:168. doi: 10.1186/s12943-022-01638-1 (PMC9392287; doi:10.1186/s12943-022-01638-1)
Supplement: Supplementary file 8 — Additional file 8: Supplementary Table S1. Sequences of primers used in RT-qPCR assay. [file 12943_2022_1638_MOESM8_ESM.docx]

**Supplementary Table S1. Sequences of primers used in RT-qPCR assay.**

| **Names** | **Sequences (5’-3’)** |
| --- | --- |
| *STEAP3-AS1*-F | GCTAGCTGCCTTTGACCTCC |
| *STEAP3-AS1*-R | TAGGGAGCTGGTGAAGGTTTG |
| *STEAP3*-F | CAGTCCTCACTGGGCTTTGT |
| *STEAP3*-R | AAGGTGGGAGGCAGGTAGAA |
| *HIF-1α*-F | ACCTATGACCTGCTTGGTGC |
| *HIF-1α*-R | GGCTGTGTCGACTGAGGAAA |
| *PGK1*-F | TGGACAATGGAGCCAAGTCG |
| *PGK1*-R | CTCCACTTCTGGGCCTACAC |
| *VEGFA*-F | CTGTCTAATGCCCTGGAGCC |
| *VEGFA*-R | ACGCGAGTCTGTGTTTTTGC |
| *SLC2A3*-F | GGTGGCTGCTTTATGGGACT |
| *SLC2A3*-R | GTAAAACCCAGTAGCAGCGG |
| *CDH2*-F | TGAAACGGCGGGATAAAGAG |
| *CDH2*-R | GGCTCCACAGTATCTGGTTG |
| *Slug*-F | AGATCTGCCAGACGCGAACT |
| *Slug*-R | GCATGCGCCAGGAATGTTCA |
| *Snail*-F | TTACCTTCCAGCAGCCCTAC |
| *Snail*-R | GCTTCGGATGTGCATCTTG |
| *VIM*-F | CGGGAGAAATTGCAGGAGGAGA |
| *VIM*-R | TCTTGGCAGCCACACTTTCAT |
| *Wnt1*-F | GCCCTAGCTGCCAACAGTAGT |
| *Wnt1*-R | GAAGATGAACGCTGTTTCTCG |
| *Wnt3a*-F | CACCACCGTCAGCAACAGCC |
| *Wnt3a*-R | AGGAGCGTGTCACTGCGAAAG |
| *Wnt5b*-F | ATGCCCGAGAGCGTGAGAAG |
| *Wnt5b*-R | ACATTTGCAGGCGACATCAGC |
| *DKK1*-F | CAGGCGTGCAAATCTGTCT |
| *DKK1*-R | AATGATTTTGATCAGAAGACACC |
| *AXIN2-*F | TGACTCTCCTTCCAGATCCCA |
| *AXIN2-*R | TGCCCACACTAGGCTGACA |
| *CCND1-*F | CACCTTATTCATGGCTGAAGTC |
| *CCND1-*R | ACAAACCTCCACTGGATGGT |
| *LGR5-*F | CACCTCCTACCTAGACCTCAGT |
| *LGR5-*R | CGCAAGACGTAACTCCTCCAG |
| *GAPDH*-F | AGACAGCCGCATCTTCTTGT |
| *GAPDH*-R | CTTGCCGTGGGTGAGTCAT |
