# Supplementary material for: Hypoxia-induced lncRNA STEAP3-AS1 activates Wnt/β-catenin signaling to promote colorectal cancer progression by preventing m6A-mediated degradation of STEAP3 mRNA
Source: Mol Cancer. 2022 Aug 19;21:168. doi: 10.1186/s12943-022-01638-1 (PMC9392287; doi:10.1186/s12943-022-01638-1)
Supplement: Supplementary file 9 — Additional file 9: Supplementary Table S2. Sequences of primers used in ChIP-qPCR assay. [file 12943_2022_1638_MOESM9_ESM.docx]

**Supplementary Table S2. Sequences of primers used in ChIP-qPCR assay.**

| **Names** | **Sequences (5’-3’)** |
| --- | --- |
| Site 1-F | TTTGGTTTGCCTGTAGAAAGCAG |
| Site 1-R | ACTTGGCCAAAGTCACACAGTC |
| Site 2-F | CAACGTACCAGGCACACTTCT |
| Site 2-R | AGGAGGACTTGCAGAGTGAGT |
| Site 3-F | CTGCAGTTGGCACCTGGTAA |
| Site 3-R | TTCCTCCCTCTGGCAACTCA |
| Site 4-F | ATGTGCTGAGTTGCCAGAGGGA |
| Site 4-R | GCTACAGCACCGCAAGCAGA |
| Site 5-F | CAGCTCGTGGACATCAGCAA |
| Site 5-R | CTGCCCATTCTGCACCATAC |
| Site 6-F | GTCCTCAGATCCCCAAGCAC |
| Site 6-R | AGCTCAGCATAGAGTAGGTGT |
| Site 7-F | GTCTGGCATTCTTTCGCCAAG |
| Site 7-R | GGGGCTAGATAATGGCTCCAC |
